# Reducing Abdominal Aortic Aneurysm Progression by Blocking Neutrophil Extracellular Traps Depends on Thrombus Formation

**DOI:** 10.1016/j.jacbts.2023.11.003

**Published:** 2024-01-10

**Authors:** Nahla Ibrahim, Sonja Bleichert, Johannes Klopf, Gabriel Kurzreiter, Hubert Hayden, Viktoria Knöbl, Tyler Artner, Moritz Krall, Alexander Stiglbauer-Tscholakoff, Rudolf Oehler, Peter Petzelbauer, Albert Busch, Marc A. Bailey, Wolf Eilenberg, Christoph Neumayer, Christine Brostjan

**Affiliations:** a Division of Vascular Surgery, Department of General Surgery, Medical University of Vienna and University Hospital Vienna, Vienna, Austria; b Division of Cardiology, Department of Internal Medicine II, Medical University of Vienna and University Hospital Vienna, Vienna, Austria; c Division of Cardiovascular and Interventional Radiology, Division of Molecular and Gender Imaging, Department of Biomedical Imaging and Image Guided Therapy, Medical University of Vienna and University Hospital Vienna, Vienna, Austria; d Division of Visceral Surgery, Department of General Surgery, Medical University of Vienna and University Hospital Vienna, Vienna, Austria; e Skin and Endothelium Research Division, Department of Dermatology, Medical University of Vienna and University Hospital Vienna, Vienna, Austria; f Department for Visceral, Thoracic and Vascular Surgery, Technical University of Dresden and University Hospital Carl-Gustav Carus, Dresden, Germany; g Leeds Institute for Cardiovascular and Metabolic Medicine, School of Medicine, University of Leeds, Leeds, United Kingdom; h Leeds Vascular Institute, Leeds General Infirmary, Leeds, United Kingdom

**Keywords:** abdominal aortic aneurysm, mouse model, neutrophil extracellular trap, thrombus

## Abstract

Neutrophil extracellular traps (NETs) are implicated in the pathogenesis of abdominal aortic aneurysm (AAA), located in adventitia and intraluminal thrombus. We compared the therapeutic potential of targeting upstream or downstream effector molecules of NET formation in 2 murine AAA models based on angiotensin II or peri-adventitial elastase application. In both models, NETs were detected in formed aneurysms at treatment start. Although NET inhibitors failed in the elastase model, they prevented progression of angiotensin II–induced aneurysms with thrombus, which resembles established human disease (including thrombus development). Blockade of upstream NET mediators was more effective than interference with downstream NET molecules.

## Highlights

• For AAA, surgical repair remains the only treatment to date, with a lack of drug options for management of aneurysm progression or rupture risk.
• NETs are implicated in the pathogenesis of cardiovascular diseases, including AAA, atherosclerosis, and thrombosis. The present study compared the therapeutic potential of distinct NET inhibitors on murine AAA by targeting major upstream or downstream effector molecules of NET formation.
• NET inhibition, in particular blockade of upstream NET mediators, prevented progression of AngII-induced aneurysms, in which treatment success was dependent on thrombus formation, which is a frequent feature of established human disease.
• These findings may also be of relevance to other NET-driven conditions and the current efforts to develop NET-targeting drugs for clinical application.

## Visual Abstract

**Figure:**
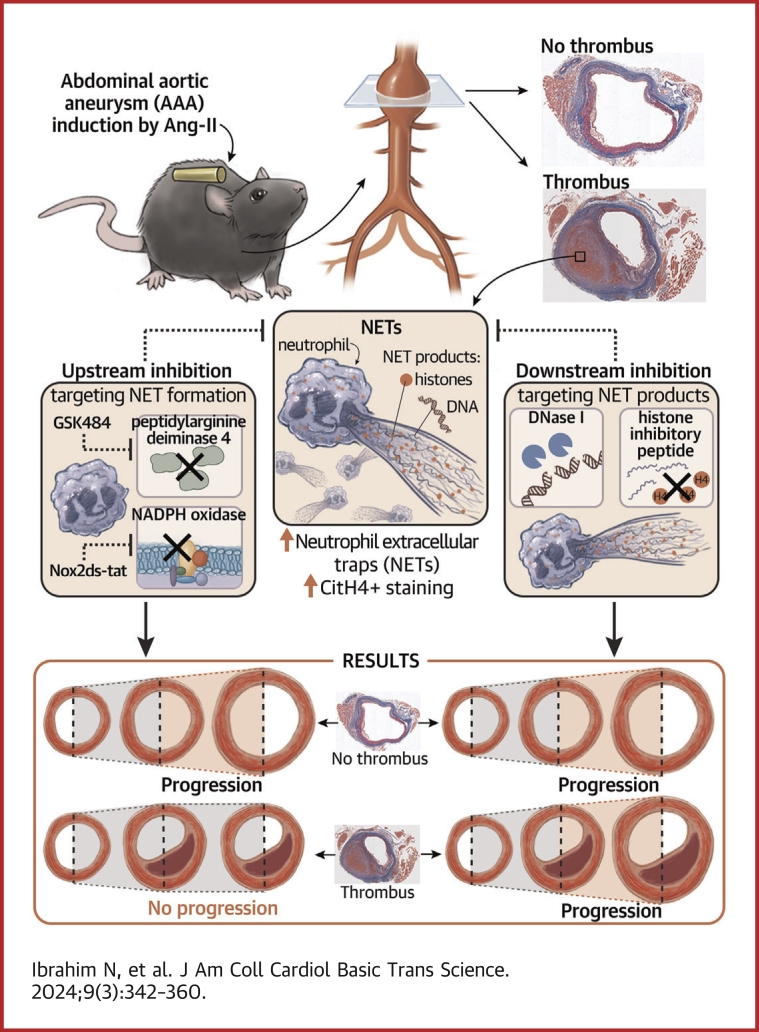

Abdominal aortic aneurysm (AAA) is a progressive dilatation of the aorta due to weakening of the wall, which is often accompanied by an intraluminal thrombus (ILT).^1^ Although mostly asymptomatic, AAAs tend to slowly grow with an eventual risk of rupture, which confers high mortality. Surgical repair remains the only treatment to date, with a lack of pharmaceutical drug options for the management of aneurysm progression or lowering of rupture risk.^2^

AAA pathology involves an interplay of endothelial cells, smooth muscle cells (SMCs), and extensive immune cell infiltration that drives vessel wall degeneration through inflammation.^3^ Neutrophils in particular are found in abundance in the adventitia and ILT.^4^ In an experimental animal model, depletion of neutrophils inhibited AAA formation.^5^ Neutrophil mediators such as myeloperoxidase (MPO), matrix metalloproteinases (MMPs), and neutrophil elastase were found to contribute to AAA development by matrix destruction.^6^^,^^7^

As part of the immune defense against pathogens, neutrophils may release their content (decondensed DNA strands, decorated with nuclear, cytosolic, and granular components such as histones, elastase, MPO, and cathepsin G) into the extracellular space as neutrophil extracellular traps (NETs).^8^ This process can be initiated via multiple pathways. In the NADPH oxidase 2 (Nox2)-dependent pathway, the production of reactive oxygen species and the nuclear translocation of MPO as well as elastase trigger chromatin decondensation.^9^ Conversely, in a Nox2-independent pathway, the activation of protein arginine deiminase 4 (PADI4) leads to histone citrullination and NET release.^10^ The pathways can be triggered separately in vitro; however, in vivo*,* they likely occur concomitantly.^11^ Of note, citrullinated histones (CitH3/CitH4) are rarely observed in other cellular processes and are therefore used as a characteristic marker of NET formation.^12^

The exposure of neutrophil DNA and intracellular proteins may have detrimental consequences, and studies have reported the presence of NETs in various diseases such as rheumatoid arthritis and cancer, where they contribute to pro-inflammatory processes.^11^ Of note, NETs have also been implicated in the pathogenesis of cardiovascular diseases and are known to promote atherosclerosis and thrombosis.^13^ Extracellular histone H4 released from NETs reportedly triggers a form of lytic cell death, whereby histone H4 mediates SMC membrane lysis.^14^ Preventing this interaction between histones and membranes by a novel histone inhibitory peptide (HIPe) averted SMC death and stabilized atherosclerotic lesions in mice.^14^ NETs also contribute to venous and arterial thrombus formation^15^^,^^16^ via the cell-free DNA acting as a scaffold for platelet and red blood cell aggregation and via histones increasing thrombin generation by activating platelets.^16^

Although most AAAs display severe atherosclerosis, several studies have investigated the specific role of NETs in AAA disease in which they were mainly observed in the adventitia and the luminal side of the ILT.^17^ Yan et al^18^ reported that NETs promote AAA development through the recruitment of dendritic cells and their type I interferon production. Administration of DNase I to dismantle NETs was effective in preventing aneurysm formation when given during the early induction phase of an AAA mouse model based on aorta elastase perfusion. Comparably, treatment with chloramidine, an irreversible pan-PAD inhibitor, significantly mitigated AAA formation in this mouse model.^19^

Because patients with AAA present with established disease, we have previously addressed the role of NETs in aneurysm progression as opposed to formation, with a particular emphasis on histone citrullination. In line with earlier reports, we found a marked deposition of CitH3 in the ILT and the aortic wall tissue of patients with AAA compared with healthy control subjects as well as in their plasma samples.^20^ Our results suggested that circulating CitH3 holds prognostic information to predict rapid aneurysm growth in patients with AAA. Moreover, we found that histone citrullination was a promising therapeutic target to block disease progression, as the specific PADI4 inhibitor GSK484^10^ prevented further AAA growth when applied to established aneurysmatic lesions.^20^ These findings were recently confirmed.^21^

We therefore hypothesized that the efficacy of blocking AAA progression might vary with the targeted pathway and molecule involved in NET formation. The aim of the present study was to compare the therapeutic impact of distinct inhibitors on established AAA disease by either blocking upstream signaling events to prevent further NET induction (via Nox2ds-tat for the Nox2-dependent pathway or GSK484 for the PADI4-dependent pathway) or by destroying and inactivating toxic components of already formed NETs (via DNase I for cell-free DNA or HIPe for extracellular histones). Two mouse models were used: aneurysms induced by angiotensin II (AngII) frequently involve an intramural thrombus and enter a progression stage resembling human established disease, whereas acute aorta injury by peri-adventitial elastase is considered a model more closely reflecting disease formation and initial expansion.^22^

## Methods

### Ethical Approval

Animal experiments were approved by the local ethics committee and the Austrian Ministry of Science (BMWFW-66.009/0355-WF/V/3b/2016, 0248-WF/V/3b/2017, and 2020-0.547.895), conforming to the European Directive 2010/63/EU and the Austrian Animal Experiment Act 2012. The experiments adhered to standard procedures of anesthesia and euthanasia as specified in the Supplemental Methods.

### AngII-Induced AAA Formation in ApoE Knock-out Mice (AngII Model)

Male mice homozygous for the ApoE mutation (ApoE knock-out, B6.129P2-ApoE^tm1Unc^/J@Him), aged 11 to 15 weeks, were kept on a normal diet. Suprarenal aneurysms were induced by subcutaneously implanted ALZET 2004 osmotic mini-pumps (DURECT Corp) releasing AngII (Bachem) at 1,000 ng/kg per minute for 28 days.^23^ Mice were monitored for aneurysm development by ultrasound at baseline (BL) (preoperatively) and day 8 and day 27 postoperatively.

### External Porcine Pancreatic Elastase (EPPE) Model for AAA Formation in Wild-Type Mice

Male C57BL6/J (wild-type) mice aged 9 to 12 weeks were given a topical peri-adventitial application of 10 μL porcine pancreatic elastase (7.6 mg/mL; Sigma-Aldrich) to the infrarenal aorta for 5 minutes.^24^^,^^25^ Aneurysms develop over 14 days in this model and are monitored by ultrasound at BL and days 4 and 13 postoperatively.

### 3-Dimensional Ultrasound Monitoring of AAA Development

The Vevo 2100 or 3100 Imaging System (FUJIFILM VisualSonics Inc) was used to record 157 frames over a 12 mm abdominal aortic stretch in a semi-automated fashion (Supplemental Methods).^25^^,^^26^ The aortic volume was calculated in cubic millimeters with Vevo LAB 5.6.1 software (VisualSonics); the maximum diameter of the aorta (inner-to-inner wall) at maximal blood flow was additionally determined from B-mode images or individual frames of the three-dimensional scan. Independent measurements by 2 observers were averaged in volume and diameter calculations.

Of note, the last experimental AAA measurements by ultrasound were conducted on the day before animal sacrifice. Hence, ultrasound data of AAA growth refer to day 13 in the EPPE model and day 27 in the AngII model, whereas tissue analyses were based on aortas harvested on days 14 and 28, respectively.

### Intravenous Drug Administration

Because treatment was to be initiated after aneurysms had formed, mice were only included in the study if aortic volume growth (in ultrasound analysis) exceeded 120% at day 8 in the AngII model or day 4 in the EPPE model. Mice with an established aneurysm were stratified into treatment groups by matching 1:1 for percent aortic volume growth with the phosphate-buffered saline (PBS) control cohort (n = 30 in the AngII model; n = 32 in the EPPE model).

The external jugular vein was catheterized on the following day (ie, on day 9 for the AngII model and day 5 for the EPPE model) for intravenous drug administration via vascular access button (VAB 1 channel 22ga; Instech Laboratories Inc), which was inserted subcutaneously as previously described.^20^^,^^23^ The NET inhibitors (or control PBS) were dispensed in a daily volume of 250 μL via the access button until the end of the experiment (n = 7-9 mice per treatment group). Drug doses are provided in the Supplemental Methods. Animals were sacrificed by a ketamine/xylazine overdose at the experimental end point (on day 28 for the AngII model and day 14 for the EPPE model), perfused with PBS^–/–^ followed by 4% paraformaldehyde, and the aortas were gently dissected and processed for paraffin embedding.

### Histology and Immunofluorescence Staining

Aorta sections (3-5 μm) were investigated for neutrophils (Ly6G) and NETs (CitH4 and DNA) by immunofluorescence staining. Consecutive cuts were analyzed for SMCs (smooth muscle alpha actin [SMA]) and macrophages (CD68) or vimentin. Staining procedures are specified in the Supplemental Methods. In addition, consecutive tissue sections were also subjected to Masson’s trichrome staining, following the manufacturer’s protocol (Polysciences).

For quantification of NETs in the time course experiments, aortic areas in the full image scans were manually marked as regions of interest using Fiji software (ImageJ, National Institutes of Health). Quantification of neutrophils and NETs was conducted with a self-created pipeline in CellProfiler 4.0.7 software (Broad Institute) in which the percentage of aortic area covered by CitH4 (NET area), the number of neutrophils (Ly6G-positive cells), and number of Ly6G-positive as well as CitH4-positive neutrophils (NETosing neutrophils) per square millimeters of aortic area were determined.

For the treatment animals, tissue scoring of neutrophils, NETs, macrophages, vimentin, and SMA was conducted manually by ordinal scoring based on 4 levels of signal frequency; that is, 0-3 for none, few, moderate, or high number of detectable cells, respectively.

### Statistical Analysis

To calculate the minimum number of experimental animals required to potentially obtain a significant outcome at a statistical power of 80% and significance of 5%, a therapeutic reduction of AAA development (maximum vessel diameter at study end) of at least 30% was predicted, and calculations were based on previously published data of AAA growth in both models.^27^ Using an independent sample Student’s *t*-test, the required sample size equaled 7 per group.

Data were plotted as individual data points with the mean ± SEM or as box plots showing the median with 25th and 75th percentiles. For AAA growth, data are expressed in percentage of aneurysm volume from BL (100%) or in maximum aortic diameter (millimeters). The Wilcoxon signed rank test was applied for comparisons within each group vs baseline, whereas the Wilcoxon rank sum test was used to compare unmatched mouse groups at the individual time points of the time course. Treatment mice were matched 1:1 for percent aortic volume growth at day 8 (AngII model) or day 4 (EPPE model) with the closest sized control mouse in the respective PBS cohort; differences between groups were evaluated with the Wilcoxon signed rank test at the experimental endpoint. For analysis of thrombus impact, treatment effect was determined for the thrombus or no thrombus groups at the experimental endpoint by using the Wilcoxon rank sum test. The Fisher exact test was used for categorical data; that is, to examine the association between NET location (thrombus or wall) with experimental time point (day 8 and day 28).

Statistical analyses were conducted with GraphPad Prism version 9.0.0 for Windows (GraphPad Software), SPSS 27.0 software (IBM SPSS Statistics, IBM Corporation), or Python version 3.11 (Python Software Foundation), and a significance level of *P* < 0.05 was applied. Because the analysis was exploratory in nature, no adjustments for multiple testing were made or considered in sample size calculation.

## Results

### 2 Mouse Models Exhibit NET Formation in the Time Course of AAA Development

Two established mouse models of AAA induction (the AngII model and the EPPE model) were investigated for NET deposition within the AAA. As previously described,^23^ 35% of AngII-infused mice experienced early aortic rupture (<10 days). In the remaining animals, aneurysms formed suprarenally over 4 weeks, as well as in the thoracic aorta, frequently accompanied by aortic dissection and intramural thrombus formation. It has previously been documented for the AngII model that medial tears occur in all mice, whereas thrombus formation strongly depends on the location; that is, hematomas are mostly observed when the ostium of the left suprarenal artery is affected.^28^

**Figure 1:**
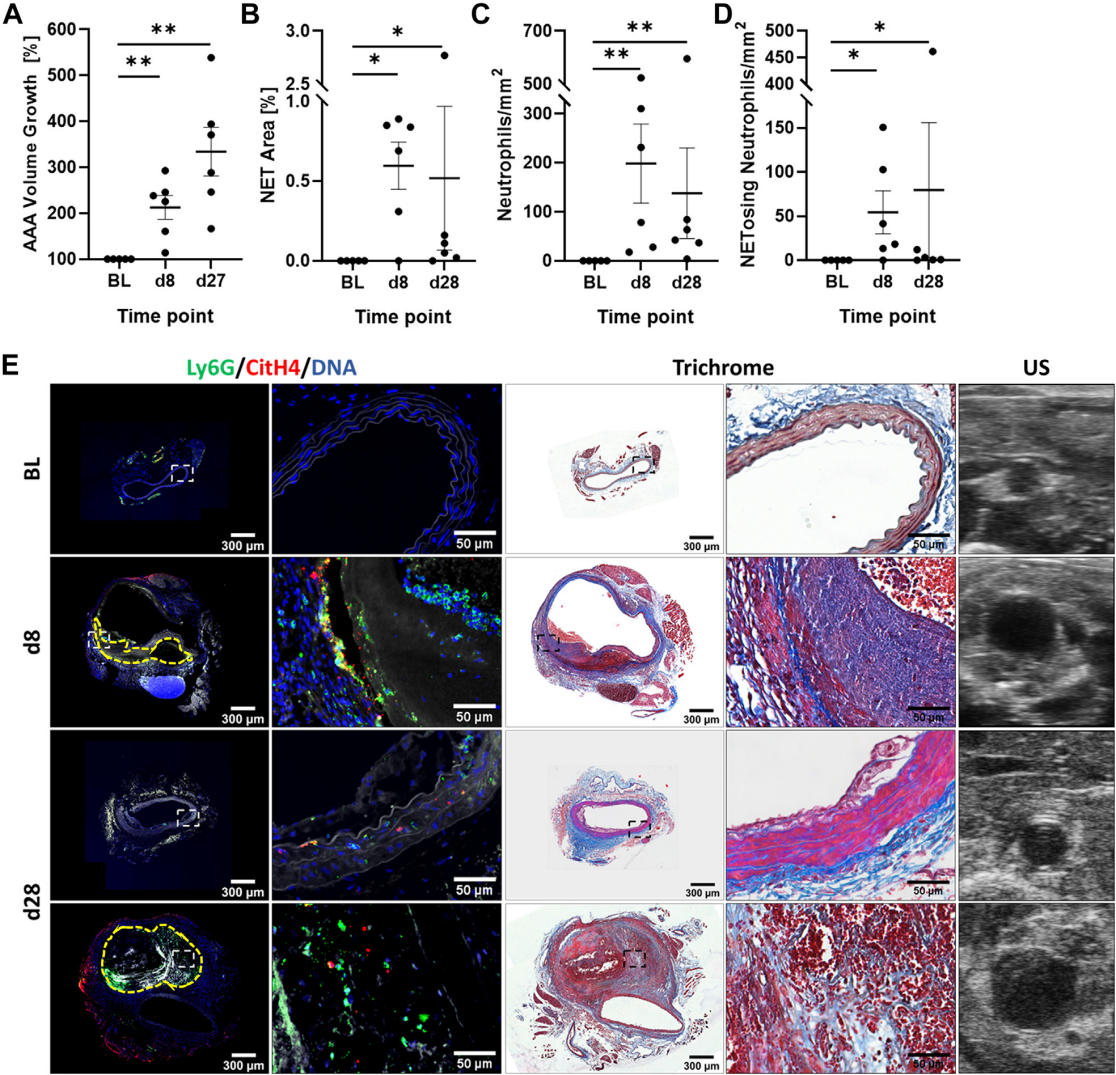
AAA Progression and NET Presence in the AngII Model (A) Abdominal aortic aneurysm (AAA) growth is expressed as percent increase of volume compared with baseline (BL = 100%). Quantification was conducted for the percentage of aortic area covered by citrullinated histone 4 (CitH4) (neutrophil extracellular trap [NET] area) (B), the number of Ly6G-positive neutrophils (C), and the number of Ly6G-positive and CitH4-positive neutrophils (NETosing neutrophils) per square millimeter of aortic area (D). (E) Suprarenal aortic sections from baseline, day 8 (d8), and day 28 (d28) were immunofluorescence stained for CitH4 (red), Ly6G (green), and DNA (blue); autofluorescence of elastin/collagen fibers is depicted in white. Consecutive sections were subjected to Masson’s trichrome stain, and representative ultrasound (US) images are given for the corresponding time points. AAA examples without or with (yellow dashed line) intramural thrombus are given for d28. Areas of interest are shown in zoomed images. N = 5 to 6 mice per time point. Values are presented as individual points with mean ± SEM (Wilcoxon rank sum test, ∗*P* < 0.05, ∗∗*P* < 0.01). AngII = angiotensin II.

**Figure 2:**
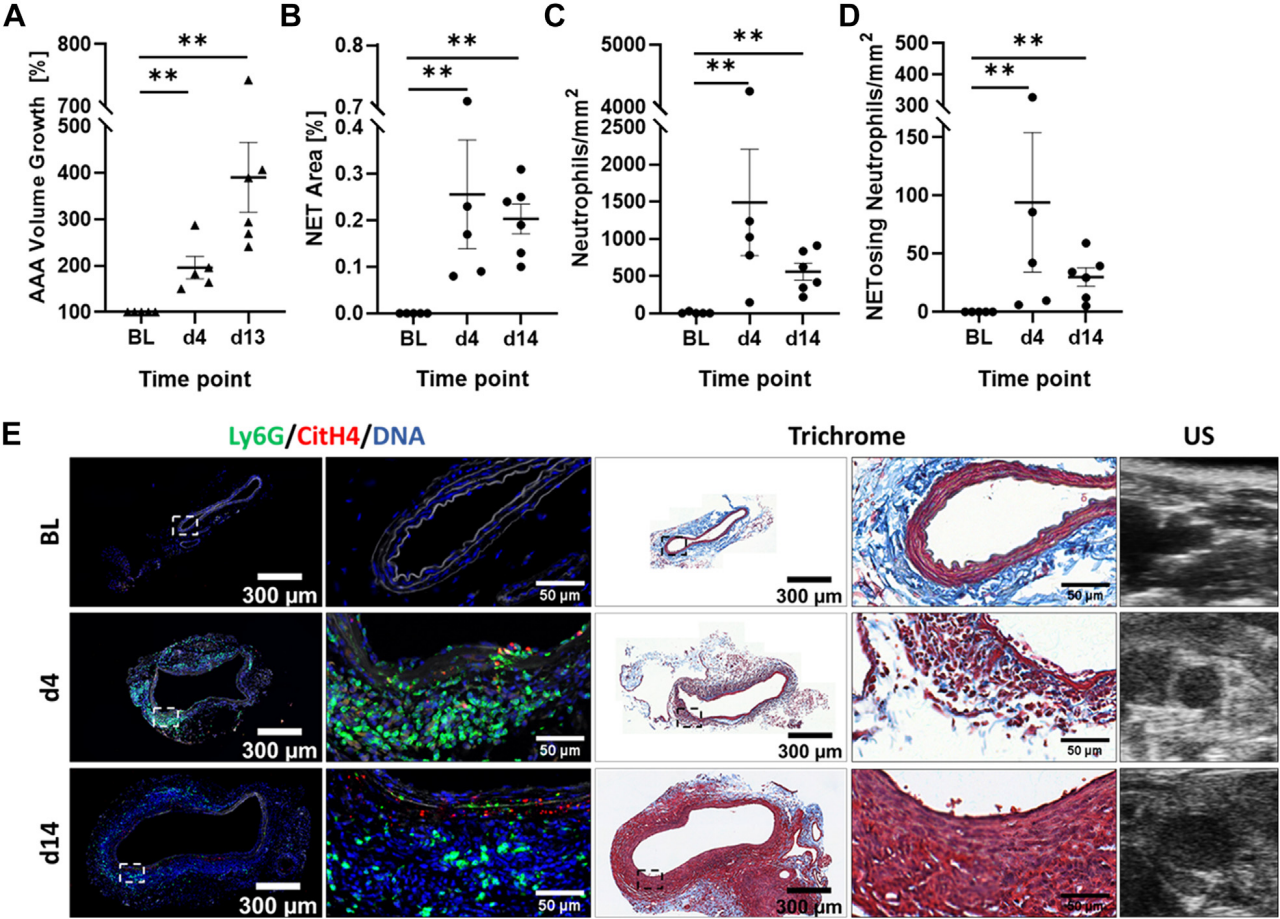
AAA Progression and NET Presence in the EPPE Model (A) AAA growth is expressed as percent increase of aortic volume from BL (BL = 100%). The percentage of NET area (B), number of neutrophils (C), and NETosing neutrophils per square millimeter of aortic area (D) were quantitated. (E) Infrarenal aortic sections from baseline, day 4 (d4), and day 14 (d14) were stained for NETs: CitH4 (red), Ly6G (green), and DNA (blue). Autofluorescence of elastin and collagen fibers is depicted in white. Consecutive sections were subjected to Masson’s trichrome stain, and representative US images are given for the corresponding time points. Marked areas of interest are shown in zoomed images. N = 5 to 6 mice per time point. Values are presented as individual points with mean ± SEM. Wilcoxon rank sum test, ∗∗*P* < 0.01; EPPE = external porcine pancreatic elastase; other abbreviations as in Figure 1.

Ultrasound measurements (Figure 1A) revealed that on day 8, the abdominal aortic volume had doubled from BL (100%) to an average of 213% ± 26.3%, *P* = 0.004 compared with BL; by day 27, the aortic volume was increased to 334% ± 53.1%, *P* = 0.004 vs BL. Comparably, aneurysms formed infrarenally in the EPPE model over 2 weeks and displayed an average 2-fold increase by day 4 in volume (196% ± 24.1%; *P* = 0.008 vs BL) (Figure 2A), which was doubled again by day 13 (390% ± 75.3%; *P* = 0.004 vs BL). No thrombus, ruptures, or aneurysm-related deaths were observed in the mice from the EPPE model.

When mice were sacrificed at the indicated time points and aorta sections were stained, neutrophils and NETs (CitH4) were essentially absent in aortas at BL for both models. In the AngII model, percent NET area (0.60% ± 0.15%), neutrophils (198 ± 81 N/mm^2^), and CitH4-positive neutrophils (NETosing neutrophils, 54 ± 24 N/mm^2^) were most prevalent at day 8 (*P* = 0.015, *P* = 0.004, *P* = 0.015 vs BL, respectively) (Figures 1B to 1E, Supplemental Figure 1). The levels decreased by day 28 (NET area 0.52% ± 0.45%; neutrophils/mm^2^ 138 ± 92; NETosing neutrophils/mm^2^ 80 ± 76) but remained elevated compared with baseline (*P* = 0.015, *P* = 0.004, and *P* = 0.015).

For mice with intramural thrombus formation, at the peak of NET prevalence (day 8), CitH4-positive staining was primarily located in the aortic wall at the interface between the thrombus and thickened remodeling adventitia, where the medial wall disruption had occurred. In contrast, at day 28, the remaining CitH4 signal was primarily located within the thrombus in all mice. Here, a significant trend was detected regarding the primary location of NETs in the vessel wall at day 8 as opposed to the thrombus at day 28 (Fisher exact test, *P* = 0.048) (Supplemental Figure 1). In the 2 mice without thrombus formation, little neutrophil or CitH4 signal was remaining at day 28.

In the EPPE model, percent NET area (0.26% ± 0.12%), neutrophils (1,488 ± 715 N/mm^2^), and NETosing neutrophils (94 ± 60 N/mm^2^) were also most abundant at the early time point of day 4 (*P* = 0.008 for all 3 parameters vs BL) (Figures 2B to 2E, Supplemental Figure 2). At the end of the experiment (day 14), the NET area (0.20% ± 0.03%), neutrophils (557 ± 113 N/mm^2^), and NETosing neutrophils (30 ± 8 N/mm^2^) remained high (*P* = 0.004 for all parameters vs BL). In histology, this model was primarily characterized by medial and adventitial thickening and elastin degradation, and areas with increased, freshly deposited collagen had a very high neutrophil infiltration at day 4, which was sustained but seen to a lesser extent on day 14. CitH4 deposition appeared to be localized to the media, where, strikingly, CitH4 was detectable in areas in which elastin fibers were completely absent or mostly broken; areas with intact elastin fibers did not show any CitH4.

Of note, in both mouse models, CitH4-positive cells negative for Ly6G were detected, suggesting that cell types other than neutrophils might contribute to extracellular trap formation. As shown in Supplemental Figure 3, Ly6G-positive cells constituted the majority of CitH4-positive cells, thus arguing for neutrophils as the predominant source of extracellular traps in the AAA models.

### Upstream NET Inhibitors Are More Effective than Downstream NET Blockade in Preventing Disease Progression in the AngII AAA Model

To test the effect of NET inhibitors on AAA progression, treatment was administered after establishment of disease. Based on an initial ultrasound assessment of 4 time points per model (data not shown), the treatment was started after day 8 for the AngII model and day 4 for the EPPE model; this is when the aortic volume growth had reached an average value of approximately 200% and when NETs were found to be present in the tissue (Figures 1 and 2).

**Figure 3:**
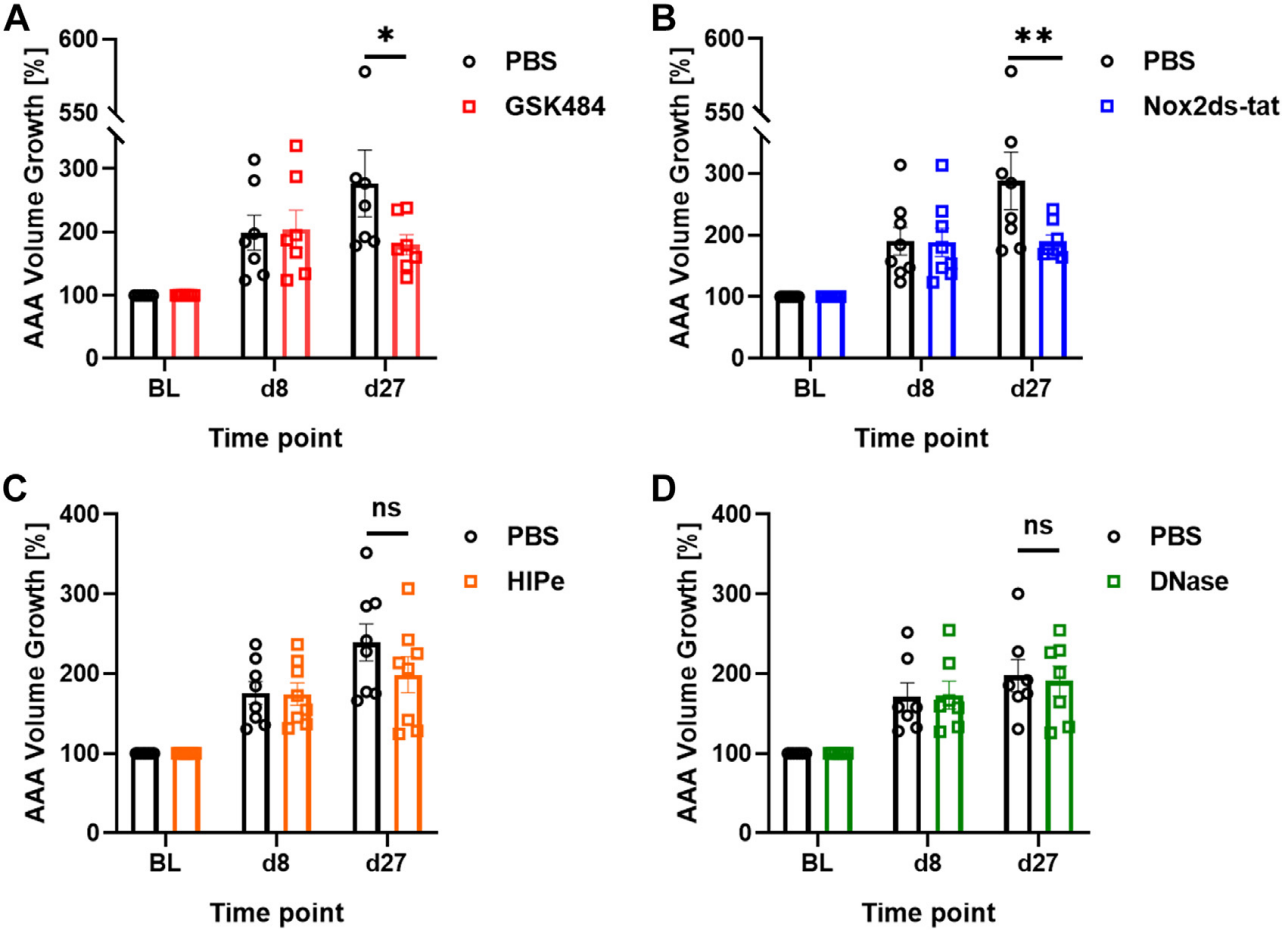
Upstream vs Downstream NET Inhibition in the AngII Model Mice of treatment and control (phosphate-buffered saline [PBS]) groups were matched 1:1 at day 8 for percent increase in aortic volume compared with BL (BL = 100%). Treatment was injected intravenously daily from day 9 to day 27 (d27). The upstream pathways of NET formation were targeted with GSK484 (n = 7) (A) and Nox2ds-tat (n = 8) (B). To inhibit the downstream products of NETs, histone inhibitory peptide (HIPe) (n = 8) (C) and DNase I (n = 7) (D) were administered. Values are presented as individual points with mean ± SEM. Group differences at d27 (Wilcoxon signed rank test) are marked by ∗*P* < 0.05, ∗∗*P* < 0.01. ns = not significant; other abbreviations as in Figure 1.

The frequency of AAA ruptures and thrombus development in the AngII cohort (Supplemental Table 1) corresponded to our previous observations.^23^ Mice were stratified into control and treatment groups based on 1:1 matching by aortic volume growth from baseline to day 8 and underwent jugular vein catheterization for daily intravenous treatment. First, GSK484 was administered to target histone citrullination. At stratification (day 8), both groups showed a comparable degree of established disease; that is, AAA growth was to 205% ± 30% (GSK484) and 199% ± 28% (PBS). The AAAs in mice that were subsequently given GSK484 treatment did not progress compared with the PBS group (mean at day 27: 180% ± 16% vs PBS 277% ± 53%; Wilcoxon signed rank test, *P* = 0.016) (Figure 3A). Maximum aortic diameter analysis agreed with volume results (Wilcoxon signed rank test, *P* = 0.047) (Supplemental Figure 4A).

To target the NADPH oxidase activation pathway of NET formation, Nox2ds-tat peptide was used.^29^ Treatment significantly attenuated aneurysm progression compared with the control (mean at day 27: 190% ± 10% vs PBS 288% ± 47%; Wilcoxon signed rank test, *P* = 0.008) (Figure 3B). When evaluating maximum aortic diameter, the results were similar (Wilcoxon signed rank analysis, *P* = 0.023) (Supplemental Figure 4B).

Downstream of the NET formation pathway, NET products were targeted to investigate their contribution to aneurysm growth. HIPe was applied to neutralize the toxic activity of cell-free histone H4,^14^ and DNase I was used to degrade the DNA of released NETs. Both downstream inhibitors failed to block further disease progression in the AngII model. For aneurysms exposed to HIPe, AAA volume growth was less pronounced but did not differ significantly from the PBS group (mean at day 27: 199% ± 23% vs PBS 239% ± 23%; Wilcoxon signed rank analysis, *P* = 0.20) (Figure 3C). The DNase I treatment failed entirely to affect aneurysm progression (mean at day 27: 191% ± 19.0% vs PBS 198% ± 20.3%; Wilcoxon signed rank test, *P* = 0.81) (Figure 3D). In line with aortic volume growth, these 2 treatment groups did not differ significantly from PBS control regarding maximum aortic diameter increase (Supplemental Figures 4C and 4D).

**Figure 4:**
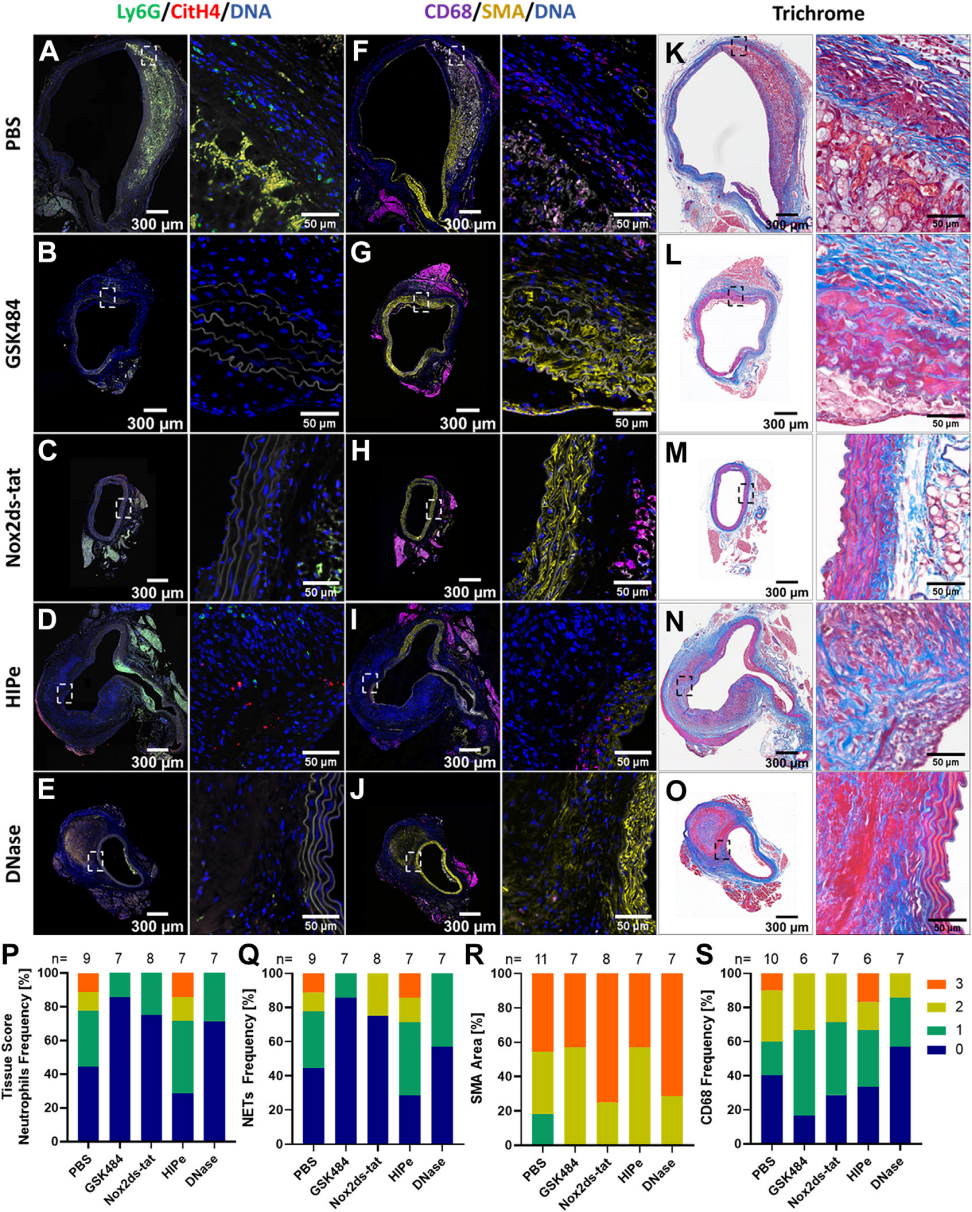
NET Accumulation at d28 in the AngII Model Suprarenal consecutive aortic sections from day 28 (d28) were stained for NETs (A-E) by CitH4 (red), Ly6G (green), and DNA (blue) immunofluorescence, while smooth muscle cells and macrophages (F to J) were visualized with smooth muscle alpha actin (SMA) (yellow), CD68 (magenta), and DNA (blue) staining; autofluorescence of elastin and collagen fibers is depicted in white. (K to O) Masson’s trichrome stain. Marked areas of interest are shown in zoomed images. Semiquantitative tissue scoring for the indicated number (n) of mice was performed for neutrophils (Ly6G) (P), NETs (CitH4) (Q), smooth muscle cells (SMCs) (SMA) (R), and macrophages (CD68) (S). Abbreviations as in Figures 1 and 3.

To assess the impact of the inhibitors on the frequency of neutrophils, NETs, SMCs, and macrophages in treated aneurysms, aortic tissue was stained for Ly6G and CitH4, SMA, and CD68, and it was scored (0-3) for semi-quantitative analysis (Figure 4, Supplemental Figures 5 and 6). In the AngII model, the infiltration of neutrophils and the deposition of CitH4 in the aortic tissue of mice administered upstream inhibitors was lower than in the PBS cohort (56% with detectable signal of Ly6G or CitH4 in the PBS group vs 14% of aortas in the GSK484 group and 25% of aortas in the Nox2ds-tat cohort). In contrast, neutrophil and NET accumulation in aneurysms of HIPe-treated mice resembled the frequency of control mice and reached a level of 71%. Of note, although no therapeutic impact of DNase I application on AAA progression was observed, it showed an effect on the NET content of treated aneurysms, which ranged at 29% of aortas with detectable Ly6G and 43% of aortas with CitH4 signal. All treatment groups showed high scores (2-3) for SMA expression, whereas only the PBS cohort had 18% of aortas with low SMC coverage. Conversely, the highest scores (3) for macrophage infiltration were recorded for the PBS and HIPe groups.

### NET Blockade Is Effective in Mice That Develop an Intramural Thrombus in the AngII Model

Because analysis of NET distribution at day 28 had revealed that NETs were primarily detected in the intramural thrombus, we investigated whether the presence of thrombus influenced the outcome in the treatment groups. In the full PBS cohort (n = 30), 11 mice did not have a thrombus, whereas 19 mice presented with a thrombus. To increase the power for statistical analysis, mice from the GSK484 and Nox2ds-tat cohorts were pooled as the effective upstream inhibitor group, with 8 mice not having a thrombus, and 7 mice with a thrombus. For the ineffective downstream inhibitor group, consisting of HIPe and DNase I treatment cohorts, 6 mice did not have a thrombus, and 9 mice had a thrombus. Because the mice were combined into the upstream and downstream inhibitor groups (n = 15 per group), we tested if the pooling itself had influenced the results (data not shown). Compared with the full PBS cohort, the upstream inhibitor group had a significantly lower aortic volume increase at day 27 (mean: 185% ± 9% vs PBS 245% ± 19%; *P* = 0.020), whereas the downstream inhibitor group showed no significant difference from the PBS group in mean aortic volume increase at day 27 (mean: 195% ± 14%; Wilcoxon rank sum test, *P* = 0.16).

**Figure 5:**
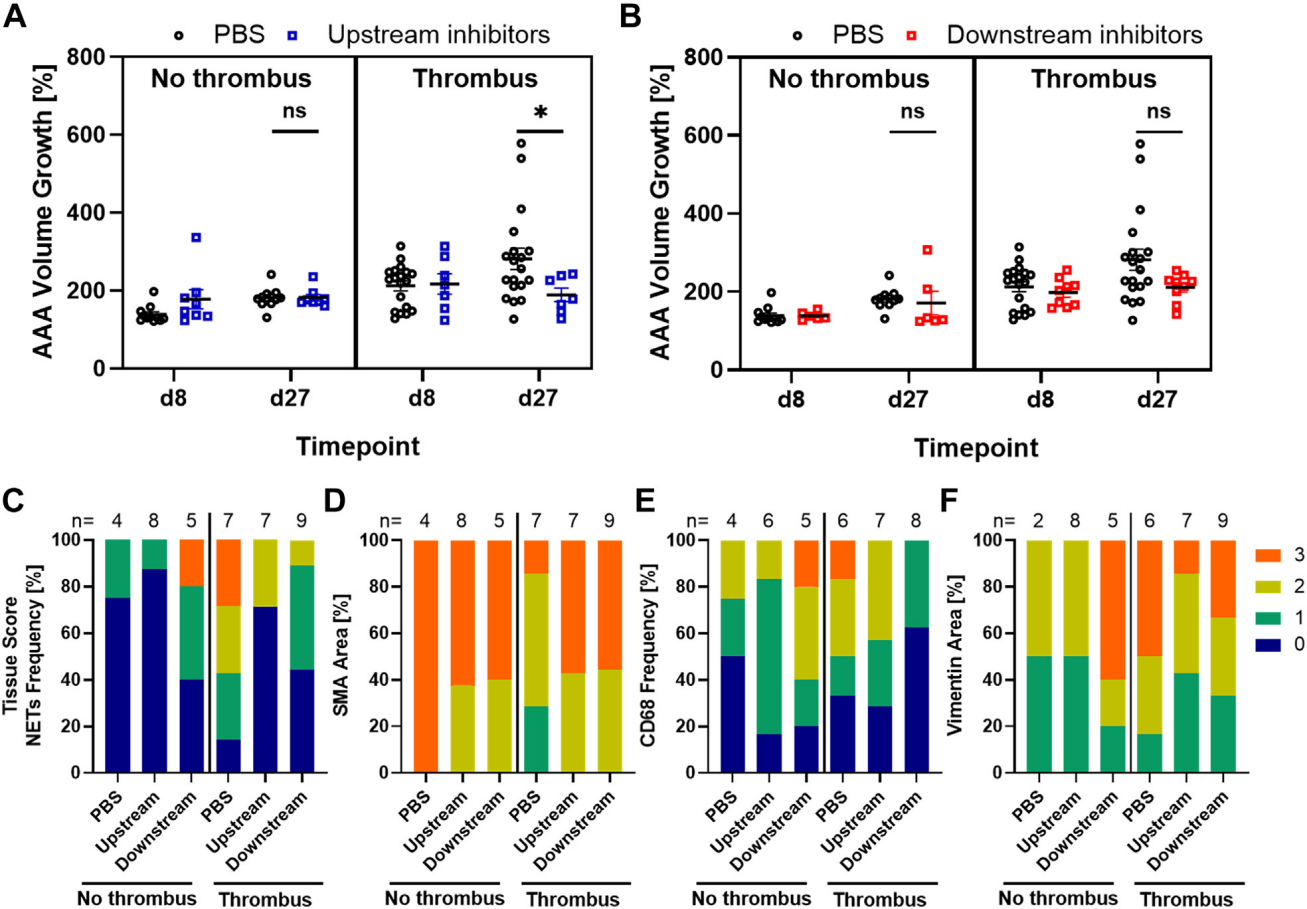
NET Inhibition in AngII Mice Without or With AAA-Associated Thrombus Mice were divided into subgroups based on intramural thrombus presence in three-dimensional ultrasound evaluation. AAA volume growth was recorded in percentage of BL (BL = 100%). Data of upstream inhibitors (GSK484 and Nox2ds-tat) were pooled (A), and data of downstream inhibitors (HIPe and DNase) were similarly pooled (B). Treatment groups were compared with the PBS cohort at d27 by using the Wilcoxon rank sum test (∗*P* < 0.05). Values are presented as individual points with mean ± SEM. Semi-quantitative tissue scoring (0-3) was performed for NETs (CitH4) (C), SMCs (SMA) (D), macrophages (CD68) (E), and vimentin (F). Abbreviations as in Figure 1, 3, and 4.

Notably, aneurysms with intramural thrombus were substantially larger than AAAs without thrombus in the PBS control group. However, both types of aneurysm significantly progressed from day 8 to day 27 (Wilcoxon signed rank test, *P* = 0.033 with thrombus, *P* = 0.003 without thrombus). The upstream inhibitor group (Figure 5A), which was effective in attenuating aneurysm growth, showed a mean aortic volume increase at day 27 significantly lower than that of the PBS controls when the thrombus subgroup was assessed (189% ± 18% vs PBS 282% ± 27%) but did not differ in the no-thrombus subgroup (182% ± 8% vs PBS 182% ± 8%). When evaluated separately for the 2 subgroups by using the Wilcoxon rank sum test, treatment effect at day 27 was significant for experimental animals with thrombus (*P* = 0.035) but not for mice without AAA-associated thrombus (*P* = 0.56). Of note, data are also given independently for the GSK484 and Nox2ds-tat cohorts in Supplemental Figure 7, and they confirmed the same tendency in mice with thrombus formation for either treatment.

The downstream NET inhibitors (Figure 5B), which were not significant in the total AngII cohort, had a lower mean aortic volume increase at day 27 of 211% ± 12% compared with PBS controls (282% ± 27%) when specifically analyzing the thrombus subgroup, showing a statistical trend for growth reduction (*P* = 0.081). There was no substantial difference in the no-thrombus subgroup (171% ± 30% vs PBS 182 ± 8%; Wilcoxon rank sum test, *P* = 0.27).

The presence of an intramural thrombus seemed to determine the efficacy of anti-NET therapy in the AngII model more markedly than the choice of upstream or downstream inhibitor. We therefore further pooled all anti-NET approaches and obtained a high significance level (*P* = 0.017) for the difference between treatment and control within the thrombus subgroup (data not shown). Again, the no-thrombus subgroup had no substantial therapeutic impact at day 27 (Wilcoxon rank sum test, *P* = 0.32).

To assess the impact of the inhibitors in relation to thrombus presence on the frequency of neutrophils and NETs, SMCs, and macrophages in treated aneurysms, a similar subanalysis was also conducted by using semi-quantitative tissue scoring (Figures 5C to 5F, Supplemental Figures 5 and 6). When focusing on mice with a thrombus, the deposition of CitH4 was lowest in the aortic tissue of animals that were given the effective upstream inhibitors compared with the PBS cohort (71% of aortas without detectable CitH4 signal vs 14% in the PBS cohort) or the downstream inhibitors, which were also effective in the thrombus cohort (44% of aortas with no detectable NETs). In terms of SMC colonization in the thrombus group, the anti-NET inhibitors showed a high SMA-positive aortic coverage (tissue scoring = 3) compared with the PBS cohort (57% in the upstream inhibitor group and 56% in the downstream inhibitor group vs 14% in the PBS group). Similarly, the frequency of vimentin (Supplemental Figures 5 and 6), an intermediate filament that is up-regulated in SMC transition from the contractile to the synthetic phenotype,^30^ was highest in the PBS group (score ≥2: 83% vs 57% in the upstream group vs 67% in the downstream inhibitor group). For macrophage infiltration in AAA with thrombus, the downstream anti-NET treatment resulted in the lowest rate while upstream inhibitors shifted the macrophage frequency toward a lower score.

### Effective NET blockade Is Associated With Reduced Vascular Remodeling in Gene Expression Analysis of AngII-Treated Mice

**Figure 6:**
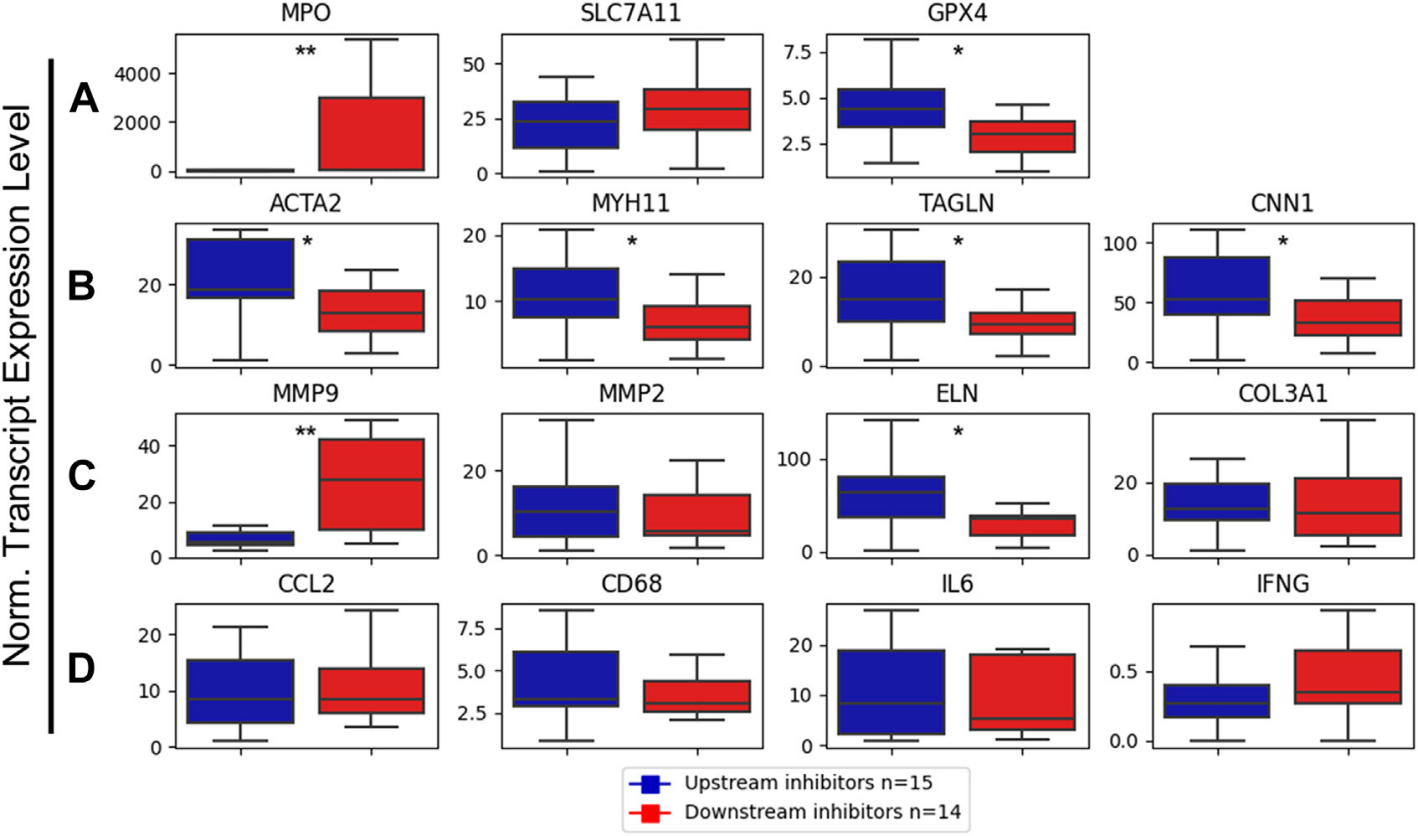
Impact of Upstream vs Downstream NET Inhibitors on Mediators of Inflammation, Matrix Remodeling, and SMC Transdifferentiation in the AngII Model Aneurysms from mice treated with upstream (n = 15) or downstream (n = 14) inhibitors of NET formation were subjected to RNA isolation, complementary DNA synthesis, and real-time polymerase chain reaction to evaluate transcript levels of genes involved in redox regulation or ferroptosis (A), smooth muscle cell differentiation (B), extracellular matrix remodeling (C), and inflammation (D). Supplemental Table 2 provides gene symbols, synonyms, and q polymerase chain reaction primer sources. Values are presented as box plots; *P* values are based on Wilcoxon rank sum test (∗*P* < 0.05, ∗∗*P* < 0.01). ACTA2 = alpha-smooth muscle actin (SMA); CCL2 = monocyte chemotactic protein 1 (MCP-1); CD68 = macrosialin; CNN1 = calponin 1; COL3A1 = collagen type III alpha 1; ECM = extracellular matrix; ELN = elastin; GPX4 = glutathione peroxidase 4; IL6 = interleukin 6; IFNG = interferon gamma; MMP = matrix metalloproteinase; MPO = myeloperoxidase; MYH11 = myosin (heavy chain) 11; SLC7A11 = solute carrier family 7 member 11; TAGLN = transgelin; other abbreviations as in Figures 1 and 4.

Although neutrophils are known to contribute to AAA formation by the production of reactive oxygen species, the secretion of inflammatory mediators, and matrix-degrading proteinases, NETs have been proposed to primarily act on SMCs via inducing transdifferentiation or ferroptosis.^31^^,^^32^ Because immunostaining analysis was semi-quantitative, we further aimed to elucidate the mechanism of effective anti-NET blockade on AAA progression by comparing beneficial upstream vs ineffective downstream NET inhibitors in tissue gene expression analysis. Aortas retrieved on experimental day 28 were subjected to RNA isolation, complementary DNA generation, and real-time polymerase chain reaction of a selected gene panel (Figure 6, Supplemental Methods).

Aneurysms that had been treated with upstream NET inhibitors showed significantly lower levels of MPO, indicating reduced neutrophil accumulation and activation (Figure 6A). Furthermore, expression of MMP-9 but not MMP-2 was substantially reduced, and elastin transcript levels were significantly higher, in AAA tissue of mice responsive to upstream vs downstream NET blockade (Figure 6C). In line with the proposed NET effect on SMC plasticity,^31^ markers of the contractile SMC phenotype such as SMA, myosin 11, transgelin, and calponin 1 were more preserved in the upstream inhibitor group (Figure 6B). A recent report further suggested that NETs promote AAA formation by inducing ferroptosis in smooth muscle cells, as evidenced by the decreased expression of cystine/glutamate antiporter solute carrier family 7 member 11 and glutathione peroxidase 4 (GPX4).^32^ Conversely, upstream NET inhibitors resulted in significantly higher transcript levels of GPX4 in aortic tissue than observed for downstream NET blockade (Figure 6A). Remarkably, parameters of inflammation such as monocyte-chemotactic protein-1, CD68, interleukin-6, or interferon gamma did not differ significantly between treatment groups (Figure 6D). When evaluated separately for mice with or without thrombus development, the described regulatory effects on gene expression in AAA tissue were predominantly observed for the thrombus group (Supplemental Figure 8). Furthermore, comparison between the 4 distinct compounds revealed a more pronounced preservation of contractile SMC genes by Nox2ds-tat than GSK484 treatment and a predominant GSK484 impact on GPX4 and interferon gamma gene expression (Supplemental Figure 9).

### NET Blockade Does Not Reduce Disease Progression in the Elastase-Triggered AAA Model

Similar to the AngII model, anti-NET treatment in the EPPE model was administered after establishment of disease (day 5) to test the drug effects on AAA progression until the endpoint at day 14. Of note, there is generally no aortic rupture and no thrombus occurrence in the elastase-induced aneurysms.

**Figure 7:**
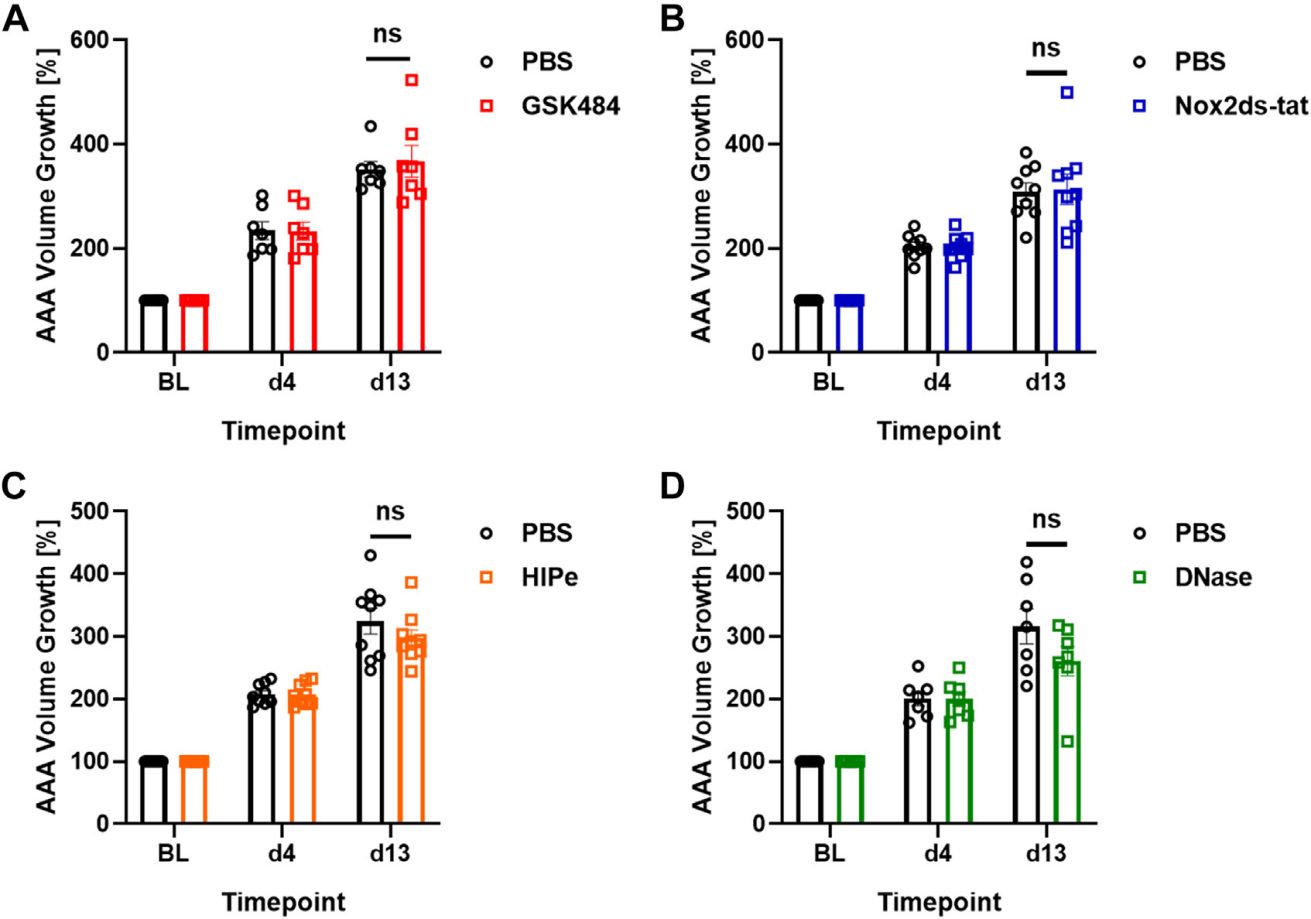
Upstream vs Downstream NET Inhibition in the EPPE Model Mice of treatment and control (PBS) groups were matched 1:1 at day 4 for percent increase in aortic volume compared with BL (BL = 100%). Treatment was injected intravenously daily from day 5 to day 13. NET formation was targeted with GSK484 (n = 7) (A), Nox2ds-tat (n = 9) (B), HIPe (n = 9) (C), and DNase I (n = 7) (D). Values are presented as individual points with mean ± SEM. Group differences at day 13 (Wilcoxon signed rank test) were not significant (ns). Abbreviations as in Figure 1, Figure 2, Figure 3, Figure 4.

None of the treatments had a significant effect in terms of attenuating aneurysm progression in the EPPE mouse model. As we showed before in a smaller cohort,^20^ GSK484 treatment was not effective in reducing aneurysm growth in this model (mean aortic volume increase at day 13: 367% ± 31% vs PBS 352% ± 15%; Wilcoxon signed rank analysis, *P* = 0.58) (Figure 7A). For the Nox2ds-tat cohort, the mean volume at day 13 was 314% ± 29% and did not significantly differ from the PBS cohort (309% ± 17%; *P* > 0.99) (Figure 7B).

The downstream inhibitors showed a slight but nonsignificant trend for reduction. The mean volume growth at day 13 for the HIPe cohort was 298% ± 13% compared with the matched PBS group (325% ± 21%; Wilcoxon signed rank test, *P* = 0.43) (Figure 7C). This trend was also reflected in the maximum diameter of the HIPe cohort with a mean of 1.18 ± 0.059 mm vs the PBS cohort (1.33 ± 0.067 mm; Wilcoxon signed rank analysis, *P* = 0.16) (Supplemental Figure 10). DNase I treatment showed a similar trend with mean aortic volume growth of 261% ± 24% vs 316% ± 28% in the PBS group (Wilcoxon signed rank analysis, *P* = 0.22) (Figure 7D). The absolute diameter, however, showed no difference between DNase and PBS treatment.

**Figure 8:**
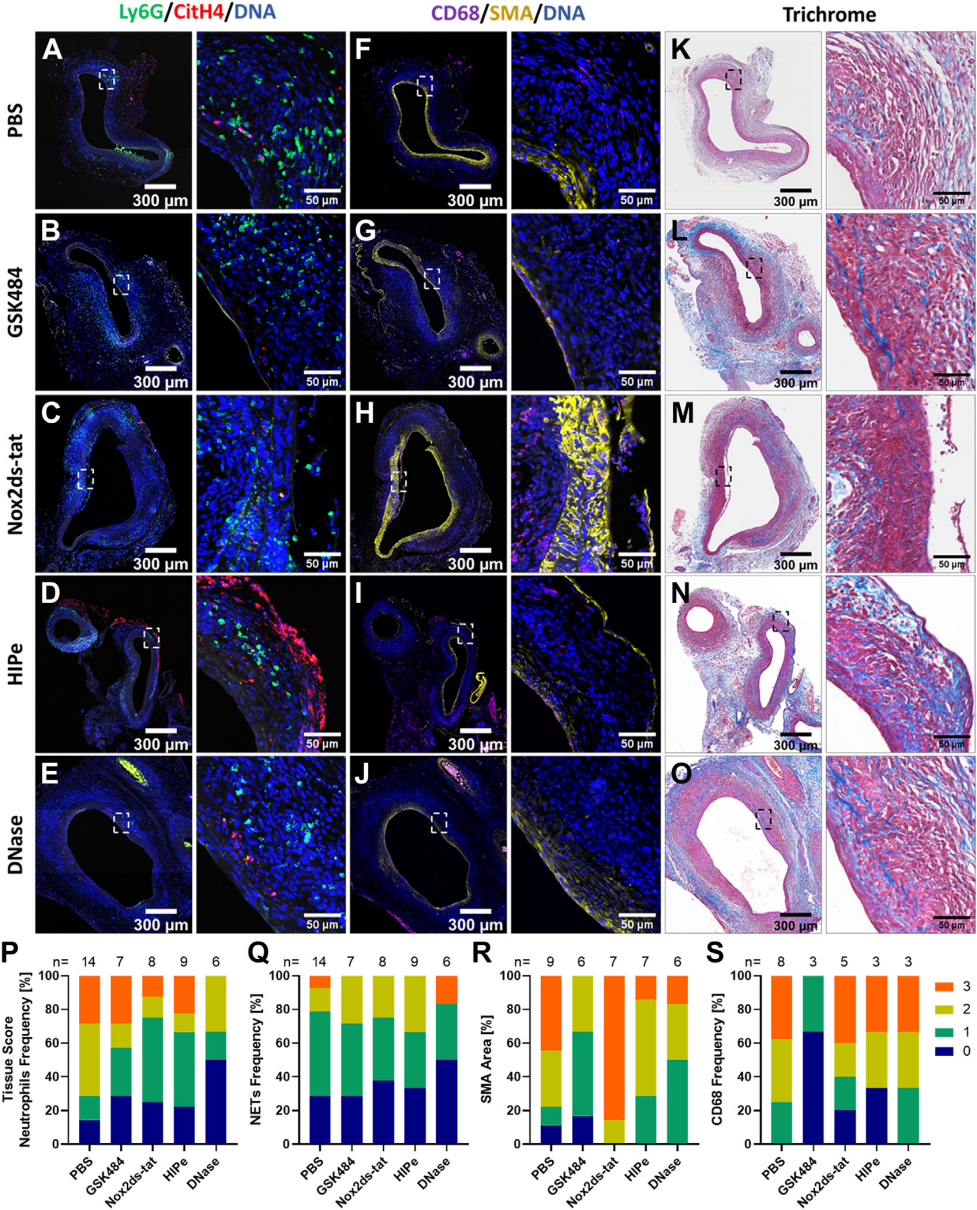
NET Accumulation at d14 in the EPPE Model Infrarenal consecutive aorta sections from day 14 (d14) were stained for NETs (A-E) by CitH4 (red), Ly6G (green), and DNA (blue) immunofluorescence, while SMCs and macrophages (F-J) were visualized with SMA (yellow), CD68 (magenta), and DNA (blue) staining; autofluorescence of elastin and collagen fibers is depicted in white. (K-O) Masson’s trichrome stain. Marked areas of interest are shown in zoomed images. Semi-quantitative tissue scoring for the indicated number (n) of mice was performed for neutrophils (Ly6G) (P), NETs (CitH4) (Q), SMCs (SMA) (R), and macrophages (CD68) (S). Abbreviations as in Figure 1, Figure 2, Figure 3, Figure 4.

Semi-quantitative tissue scoring (Figure 8, Supplemental Figure 11) revealed that GSK484, Nox2ds-tat, and HIPe treatment resulted in a similar neutrophil and NET deposition pattern in aneurysms as observed for the PBS control, with 86% of aortas in the PBS cohort showing any detectable Ly6G signal (NETs in 71% of aortas), 71% in the GSK484 cohort (NETs in 71% of aortas), 75% in the Nox2ds-tat cohort (NETs in 63% of aortas), and 78% in the HIPe cohort (NETs in 67% of aortas). Only the DNase I group showed a moderate reduction of neutrophils and NETs that was only detectable in about 50% of the aortas. Regarding SMC coverage, Nox2ds-tat showed the highest SMA-positive area (tissue scoring ≥2: 100% aortas vs 78% in the PBS cohort vs 83% in the GSK484 cohort vs 71% in the HIPe cohort vs 50% in the DNase I cohort). Macrophage infiltration was lowest in the GSK484 group (33% aortas with detectable CD68 signal), followed by the HIPe cohort (67%), then the Nox2ds-tat cohort (80%), and the PBS and DNase I cohorts having detectable signal in all aortas.

## Discussion

Previous preclinical studies have suggested NETs as a potential target in AAA disease, in which administering NET inhibitors before aneurysm induction mitigated the development of AAA.^18^^,^^19^ Comparably, we have recently shown that inhibition of NET formation by targeting histone citrullination was effective in controlling established AAA disease in an AngII model.^20^ Here, using 2 mouse models reflecting different aspects of AAA, we expanded on this therapeutic approach by targeting distinct pathways of NET formation and the toxic molecules released in the process. We found that NETs were present at the early time point (at treatment start of established disease) in both the AngII and EPPE mouse models. Of note, NETs declined more markedly in the AngII model than in the EPPE model toward the experimental endpoint and accumulated in the intramural thrombus in AngII-treated mice. However, anti-NET treatment was found more potent in halting aneurysm growth in the AngII model than in the EPPE model. There was a strong effect of upstream NET inhibition (blocking the PADI4- or Nox2-dependent pathways of NET formation) in attenuating further AAA progression in the AngII model, in particular in mice that had developed dissection and intramural thrombus. In contrast, in the EPPE model, the downstream inhibitors (neutralizing cell-free DNA or extracellular histones) exhibited a trend for reduction of aortic growth, albeit nonsignificantly. Although analysis of AAA development was primarily based on three-dimensional ultrasound-derived volume increase, which we have previously reported to be highly sensitive in aneurysm monitoring,^25^ results for maximum aortic diameter were generally in good agreement.

In the EPPE model, AAA develops because of an acute inflammatory insult and then continues to a fibrotic tissue remodeling phase, which differs considerably from the systemically induced, more chronic pathogenesis of the AngII model.^27^^,^^33^ A recent publication highlighted the similarities between human established AAA disease and the AngII model, with clear genomic parallels in terms of immune response and metabolic switching, despite the morphologic disparities of human ILT formation as opposed to wall dissections and intramural thrombi in the AngII model; in contrast, the elastase model was classified as a disease initiation model, with normalization of genomic responses by the endpoint at day 14.^22^ Similarly, NETs might contribute more to the initiation phase than the progression phase of the EPPE model, which may explain the weak impact of anti-NET therapy on established disease that we observed for the EPPE model. A previous study based on a similar AAA mouse model of elastase perfusion comparably reported that DNase I treatment was only effective in preventing aneurysm formation when given during the first 3 days of AAA induction.^18^

Regarding neutrophil infiltration and NET deposition in AAA tissue, both models peaked at the early time point of established disease; that is, when AAA volume reached about 200% at day 4 in the EPPE model and day 8 in the AngII model. Of note, the average number of infiltrating neutrophils (per square millimeters) was about 7.5-fold higher in the EPPE model, whereas the percentage of NETosing neutrophils within total neutrophils (5.2%) and NET coverage (0.26%) was substantially lower in the AAA tissue of EPPE mice compared with AngII-treated animals (29.5% and 0.60%, respectively). Thus, the particularly high frequency of NETosing (among total) neutrophils and NET (CitH4) deposition might indicate a more crucial role of NETs in the AngII model at the time of treatment initiation. However, neutrophil and NET burden decreased to almost baseline at the experimental endpoint in the AngII model (day 28) but were still detectable in the EPPE model at day 14. Importantly, we observed that in AngII mice with an intramural thrombus, NETs were deposited primarily in the aortic wall at the early time point, at the interface to the thrombus where the medial layer was disrupted and elastin degradation was observed. In contrast, at the endpoint, any remaining detectable CitH4 signal was primarily located within the thrombus.

In human AAA disease, the ILT plays a role in mechanically stabilizing the aneurysm by decreasing peak wall stress.^34^ It was also found to be a predictor of AAA growth^35^ and to increase the risk for rupture through dedifferentiation and apoptosis of SMCs by promoting degradation of the extracellular matrix and by increasing the number of infiltrating inflammatory cells.^36^ This observation is in agreement with our published findings in human AAA aortic tissue: we found high levels of neutrophil and NET deposition in both the adventitia and the ILT of human aneurysms. When CitH3 was measured in conditioned medium of fresh-frozen tissue (n = 27 per group), the levels ranged at a median of 50 ng/mg in the AAA wall (vs 1.5 ng/mg in healthy aortic tissue, *P* < 0.001) and at 629 ng/mg in the ILT (*P* = 0.002 vs the AAA wall).^20^

NETs have been previously implicated in thrombosis,^16^^,^^37^ and the accumulation of NETs in the thrombus in our human and murine aneurysmatic aorta samples supports a potential involvement in pathogenesis. Importantly, we could confirm this functional role; that is, these data showed that NET inhibition was effective in regulating AngII-induced aneurysm growth, in particular in mice that developed an intramural thrombus. The comparison of upstream and downstream NET inhibitors in the AngII model further revealed that inhibiting the NET induction process was more effective than neutralizing or dismantling already formed NET products. Although the downstream NET inhibitors failed to achieve a significant reduction of aneurysm size in statistical analysis based on the combination of AngII-treated mice with and without thrombus, a subgroup analysis revealed a moderate therapeutic effect in mice with thrombus compared with the PBS control. However, the protective effect of targeting already formed NETs was lower than for NET prevention. Whether NETs promote the thrombus formation and thereby enhance the local accumulation of tissue-destructive components, or whether the formation of a thrombus allows for prolonged NET deposition and NET-mediated vascular toxicity to drive AAA progression, remains to be determined. Of interest, a direct effect of NETs on SMC plasticity has recently been proposed.^31^

Clonal expansion of SMCs and phenotypic switching from a contractile to a synthetic and more phagocyte-like phenotype has previously been observed during the development of AAAs (and the AngII mouse model). Medial SMCs were reported to clonally expand into the adventitia and hemorrhagic areas and undergo down-regulation of differentiation markers such as SMA while up-regulating markers such as vimentin and CD68.^38^ NETs in AAA have been suggested to promote SMC switching from a contractile to a synthetic phenotype via the Hippo-YAP pathway.^31^ Our immunofluorescence results of mice with a thrombus are in agreement with this notion (ie, a lower number of SMA-positive cells were observed in the PBS vs treatment groups). However, the highest frequency of vimentin-positive cells as well as high levels of CD68-positive cells were detected in the absence of anti-NET treatment in vicinity to or within the remodeling thrombus itself. Taken together, these results suggest an effect of NETs on SMC (de-)differentiation in association with the hemorrhagic thrombus area.

When further comparing gene expression in aneurysms with successful upstream vs ineffective downstream NET blockade in AngII-treated mice, it became more evident that SMC plasticity is indeed a prime target of NET regulation in AAA. Transcript levels of the contractile SMC phenotype were significantly preserved by the upstream inhibitors GSK484 and Nox2ds-tat compared with the downstream effector molecules HIPe and DNase I. Of note, the recently proposed ferroptosis induction in SMCs by NETs, as evidenced by decreased GPX4 messenger RNA levels,^32^ was more prominent in downstream than upstream anti-NET approaches. Little difference between the treatment groups was observed for transcript levels of monocyte/macrophage markers, whereas genes expressed by neutrophils (eg, MPO, MMP-9) were more potently reduced in upstream NET blockade. Of note, the described gene regulation pattern was mainly recorded for mice with thrombus formation.

Regarding clinical translation, it should be noted that the 2 upstream inhibitors performed comparably in blocking AAA progression in the AngII model. However, the targeted approach of PADI4 inhibition might be preferable in clinical application, thereby avoiding potential unwanted side effects that are associated with Nox2 inactivation.^39^ In contrast, human genomic studies have indicated that loss of PADI4 does not lead to substantial impairment (ie, arguing for a safe application of PADI4 inhibitors).^40^ Of note, the first established PADI inhibitors (eg, F- and Cl-amidine) bound irreversibly and with similar potency to several PADI family members, including PADI4, and recognized the calcium-activated enzyme form.^41^ Among the second-generation compounds, GSK484 shows high selectivity for PADI4 and binds to the calcium-deficient protein in a reversible manner.^10^ Therapeutic interference with several family members may be preferred in conditions such as cancer or rheumatoid arthritis in which several PAD enzymes are involved.^41^ However, NET function in cardiovascular disease and the central role of PADI4 in NET induction argue for a targeted approach by selectively blocking PADI4.

### Study Limitations

CitH4 was used as a marker of NET formation because it is rarely observed in other biological processes. However, citrullinated histones only represent the PADI4 pathway of NET induction. Although alternative pathways have been reported, there are no specific markers for the, for example, Nox2-dependent activation of NET formation.

The present analysis focused on neutrophils. However, other immune cells certainly play an essential role in AAA pathogenesis^3^; in particular eosinophils, basophils/mast cells, and monocytes/macrophages are also capable of extracellular trap formation.^42^ Although 10% to 30% of CitH4-positive Ly6G-negative cells were detected in aneurysms at treatment start, the contribution of these non-neutrophil sources of extracellular traps has not been selectively addressed in this study but would be represented in the deposited CitH4 levels per square millimeter of investigated AAA area. Furthermore, inhibition of PADI4 or Nox2 reportedly also blocks extracellular trap formation by these other cell types.^43^, ^44^, ^45^, ^46^, ^47^

Of note, a possible cross-talk between PADI4 and Nox2 and hence mutual interference by GSK484 and Nox2ds-tat cannot be excluded and has been addressed by several in vitro studies, with highly controversial results. Although GSK484 does not seem to block the Nox2-dependent pathway of NET release,^48^ PADI4 inhibition by GSK484 was proposed to enhance reactive oxygen species release by Nox2.^49^ In contrast, others reported that PADI4 is physically associated with Nox2 subunits and GSK484 disrupts the complex, thereby reducing Nox2 activity.^50^

In addition, it should be noted that GSK484 and HIPe can be considered specific anti-NET treatment approaches as they are unlikely to substantially affect other physiological or pathologic processes. In contrast, DNase I and Nox2ds-tat will not only interfere with NETs but exhibit a broader impact on DNA released by other mechanisms or on additional cell types and functions regulated by Nox2, respectively. However, the observation that both upstream inhibitors GSK484 and Nox2ds-tat outperformed the downstream-acting factors (HIPe and DNase I) in AAA growth inhibition argues for a predominance of drug action via NET regulation.

## Conclusions

The findings of this study indicate that in AAA, there is a total “NET impact” on disease progression that is composed of both newly forming NETs and the deposited toxic components of NETs. As shown by the distinct outcomes of the different treatments in each of the AAA models, the balance between the contributions of either arm may depend on the pathologic trigger and the chronic setting in advanced disease. Although a combination of both an upstream and downstream inhibitor might be even more effective, a wider spectrum of side effects would also be expected, thus arguing for a targeted approach. Importantly, our analysis reveals that the thrombus in particular, which plays a crucial role in AAA progression and rupture, presents as the prime site of action for NET inhibitors in the pharmaceutical management of AAA.

Perspectives **COMPETENCY IN MEDICAL KNOWLEDGE:** Although NETs have previously been shown to contribute to the development of AAAs, our study has specifically addressed the role of NETs in disease progression, which resembles established human disease and thus highlights the potential for clinical translation. The results from our preclinical study indicate that blocking NET formation holds substantial promise for controlling aneurysm progression, which would meet a long-standing need for a pharmaceutical drug option for patients with AAA.

**TRANSLATIONAL OUTLOOK:** NETs have been identified as a potential therapeutic target in various acute and chronic diseases. To our knowledge, this study is the first to compare the efficacy of anti-NET approaches targeting upstream vs downstream effector molecules. Both the dependence of therapeutic impact on the formation of a local thrombus as well as the superior efficacy of upstream NET inhibitors may thus be of interest and relevance to other NET-driven conditions and the current efforts to develop NET-targeting drugs for clinical application.

## Funding Support and Author Disclosures

This research was funded in whole by the Austrian Science Fund (FWF) [F 5409-B21 issued to Dr Brostjan]. For the purpose of open access, the author has applied a CC BY public copyright license to any Author Accepted Manuscript version arising from this submission. Dr Bailey is personally supported by the British Heart Foundation (FS/18/12/33270) and Ms Knöbl by the DocFund program (DOC 59-833) of the Austrian Science Fund. The funders had no role in study design, data collection and analysis, decision to publish, or preparation of the manuscript. The authors have reported that they have no relationships relevant to the contents of this paper to disclose.
