# Supplementary material for: Reducing Abdominal Aortic Aneurysm Progression by Blocking Neutrophil Extracellular Traps Depends on Thrombus Formation
Source: JACC Basic Transl Sci. 2024 Jan 10;9(3):342–60. doi: 10.1016/j.jacbts.2023.11.003 (PMC10978405; doi:10.1016/j.jacbts.2023.11.003)
Supplement: Supplemental Material [file mmc1.pdf]

SUPPLEMENTAL APPENDIX

Reducing Abdominal Aortic Aneurysm Progression by Blocking Neutrophil Extracellular Traps Depends on Thrombus Formation

Nahla Ibrahim, MSc, Sonja Bleichert, MSc, Johannes Klopf, MD, Gabriel Kurzreiter, Hubert Hayden, MSc, Viktoria Knöbl, MSc, Tyler Artner, MSc, Moritz Krall, BSc, Alexander Stiglbauer-Tscholakoff, MD, Rudolf Oehler, PhD, Peter Petzelbauer, MD, PhD, Albert Busch, MD, PhD, Marc A. Bailey, MD, PhD, Wolf Eilenberg, MD, PhD, Christoph Neumayer, MD, PhD, Christine Brostjan, PhD

List of Contents

Supplemental Methods 2

Supplemental Tables 7

Supplemental Figures 10

Supplemental References 24

Supplemental Methods

Mouse anesthesia

Animal experiments were approved by the local ethics committee and the Austrian Ministry of Science (BMWFW-66.009/0355-WF/V/3b/2016, 0248-WF/V/3b/2017, and 2020-0.547.895), conforming to the European Directive 2010/63/EU and the Austrian Animal Experiment Act 2012. For all surgical procedures, mice were kept under anesthesia at 1.8-2% isoflurane in 2 L/min O_2, and administered 2.5% buprenorphine at 10 µL/g mouse subcutaneously. Post-operation (post-op), mice were administered 10% glucose at 10 µL/g mouse subcutaneously and allowed to recover under heating lamps. All mice received 7.5 mg piritramide and 0.5% glucose in 200 mL drinking water for 3 days post-op. For euthanasia, animals were injected with an overdose of approximately 100 mg/kg ketamine and 5 mg/kg xylazine intraperitoneally.

3D ultrasound scanning of mouse aortas

Mice were anesthetized and kept at 1.8-2% isoflurane and 2 L/min O_2. The Vevo 2100 or 3100 Imaging System (FUJIFILM VisualSonics Inc., Toronto, ON, Canada) with the MS550 or MX550D transmitter was used with the following settings: gain 30 dB, image depth 9.0 mm, image width 8.0 mm. Respiratory gating was set to 25% delay and a window of 50%, and the electrocardiogram trigger was set to T1 50 ms. After localization of the aorta and the left renal artery, the transmitter was moved 6 mm cranially (suprarenal scan for Ang-II model) or caudally (infrarenal scan for EPPE model), and images were acquired over 12 mm with a step size of 0.076 mm, equaling 157 frames, to produce a 3D image as we have previously described.[1,2] Mice were divided into thrombus or no-thrombus subgroups based on the identification of an intramural

thrombus by visual observation of hypoechoic or anechoic regions within the aortic wall in ultrasound images at day 8 or day 27.

For the time course experiments, the Ang-II model mice were monitored by ultrasound at baseline (BL: pre-op, n=5), day 8 (n=6), and day 27 post-OP (n=6). In the EPPE model, mice (n=5/time point) were monitored by ultrasound at BL, day 4, and day 13 post-OP. Animals were euthanized on the indicated time points or the following day for the last time point per experimental setup (i.e. on day 28 for Ang-II treated mice and on day 14 for EPPE mice).

Drug dosing in AAA mouse models

The NET inhibitors (or control PBS) were dispensed in a total volume of 250 µL PBS$^{-/-}$ (phosphate-buffered saline without calcium and magnesium) via the vascular access button from day 9 in the Ang-II model and day 5 in the EPPE model (n=7-9 mice per treatment group) till the end of the experiment at the following drug concentrations: 0.2 µg/g/d for GSK484 (Cayman Chemical, Ann Arbor, MI, USA) in the Ang-II mice,[3] and 0.4 µg/g/d in the EPPE model,[3] 3 µg/g/d for Nox2ds-tat (AnaSpec, Fremont, CA, USA), 4 µg/g/d for HIPe (Pepscan, Lelystad, Netherlands), and 7.5 µg/g twice per day for DNase I (Roche, Sigma-Aldrich) in both models. Treatment doses for intravenous injections were either based on existing literature,[4–7] or a new dose was inferred from a dose-finding study with highest tolerable dose selected for treatment *(data not shown)*. In cases where catheter patency was lost during the treatment period, drug administration was continued by intraperitoneal injection at the same dosing schedule.

Histology and immunofluorescence staining

Harvested mouse aortas were fixed overnight in 4% paraformaldehyde and processed for paraffin embedding. Aorta tissue (suprarenal for Ang-II model, and infrarenal for the EPPE model) was

sectioned at 5 μm for immunofluorescence and 3 μm for trichrome staining. For immunofluorescence detection of NETs and distinct cell populations in the resected mouse aortas, the sections were deparaffinized in xylene and subjected to decreasing concentrations of ethanol for rehydration before antigen retrieval in 5% citrate buffer (Thermo Fisher Scientific, Waltham, MA, USA).

For NET analysis, aortas were blocked with 5% bovine serum albumin in PBS and then stained for NETs with rat anti-mouse Ly6G for neutrophils (clone 1A8, 1:1000 dilution, BioLegend, San Diego, CA, USA), rabbit polyclonal anti-CitH4 antibody (07-596, 1:500 dilution, Merck), and secondary antibodies: Cy3-conjugated donkey anti-rat IgG (Dianova, Barcelona, Spain) and Alexa Fluor 647-labeled donkey anti-rabbit IgG (Thermo Fisher Scientific) at 1:400 dilution, as well as Hoechst 33342 (1:1000) for DNA.

Consecutive aorta cuts were permeabilized with 0.1% Triton X-100 in blocking solution and stained for macrophages with rat anti-mouse CD68 (clone FA-11, 1:50 dilution, Bio-Rad Laboratories, Inc., Hercules, CA, USA) and donkey anti-rat IgG DyLight 650 cross-adsorbed secondary antibody (1:200 dilution, Invitrogen, Thermo Fisher) as well as for smooth muscle cells by mouse anti-mouse Cy3-conjugated α-smooth muscle actin, SMA (clone 1A4, 1:500 dilution, Merck) and by Hoechst 33342 for nuclei visualization.

Additionally, consecutive aorta sections were also subjected to Masson's trichrome staining, following manufacturer's protocol (Polysciences, Warrington, PA, USA). Selected tissues were also stained for vimentin (H-84, 1:100, Santa Cruz Biotechnology, Inc., Dallas, Texas) including Alexa Fluor 647-labeled donkey anti-rabbit IgG (Thermo Fisher Scientific) at 1:400 dilution, as well as α-SMA antibody and Hoechst as described before.

Fluorescence images of the aortas were acquired using an automated Axio Observer Z1 microscope (Carl Zeiss MicroImaging, Inc., Oberkochen, Germany) with a 20x objective and the TissueFAXS scan software (TissueGnostics GmbH, Vienna, Austria). Brightfield images were obtained and processed using the Vectra Polaris automated slide scanner with a 20x objective (PerkinElmer, Inc., Hopkinton, MA, USA) and the Phenochart 1.0.12 software (PerkinElmer) and QuPath v0.3.2 open-source software (University of Edinburgh, UK).[8] Quantification of cells and co-localization of CitH4 and Ly6G signals were conducted with Halo 3.5.3577 (Indica Labs, Albuquerque, NM, USA) using the HighPlex FL v4.2.3 algorithm for detection.

RNA isolation, cDNA synthesis and real-time PCR

RNA was isolated from formalin-perfused aortic tissue pieces with Monarch® Total RNA Miniprep Kit (New England Biolabs, Ipswich, MA, USA). Tissue was stored at -80°C until use and was homogenized with CK14 tubes (Bertin Technologies, Montigny-le-Bretonneux, France) in a total volume of 300 µl 1x DNA/RNA Protection Reagent (RNA kit component) using Precellys 24 tissue homogenizer (Bertin Technologies) equipped with a Cryolys refrigerated module. Tissue homogenates were processed according to the RNA kit instructions including an additional heating step at 65°C for 15 minutes after proteinase K digestion to allow for reversal of formalin-based RNA modifications.[9] Nucleic acids were subjected to on-column DNase I digestion as recommended by the Monarch® Total RNA Miniprep Kit. RNA was eluted from microspin columns with 30 µl of nuclease-free water (kit component).

RNA was quantified with an Implen NP80 Nanophotometer (Implen, Munich, Germany) and assessed for RNA integrity number (RIN) with an Agilent RNA 6000 Pico Kit (Agilent Technologies, Santa Clara, CA, USA) on an Agilent 2100 Bioanalyzer (Agilent Technologies).

A total of 120 ng of RNA were transcribed into complementary DNA (cDNA) with High-Capacity cDNA Reverse Transcription Kit (Thermo Fisher Scientific) according to the manufacturer's recommendations.

Target genes and reference (housekeeping genes HPRT1 and TBP) were amplified with a 7500 Fast Real-Time PCR System (Thermo Fisher Scientific), i.e. 3 ng of cDNA were subjected to the reaction in a total volume of 20 µl. SYBR™ Select Master Mix (Thermo Fisher Scientific) was used in combination with primer pairs (200 nM final concentration for forward and reverse primer each) amplifying HPRT1, TBP, MMP9, IL6, ELN, COL3A1, MYH11, TAGLN and CNN1, while TaqMan™ Universal PCR Master Mix (Thermo Fisher Scientific) was used with commercially available TaqMan™ Assays for ACTA2, MMP2, CCL2, SLC7A11, GPX4, IFNG, CD68 and MPO. Melting curve analysis was included for SYBR based primer pairs. Primer references are listed in *Supplemental Table 2*.

Efficiency of SYBR based primer pairs was assessed with serial dilutions of template DNA, while efficiency of TaqMan™ Assays was set to 2.0. Values for each target gene were normalized separately to the reference genes HPRT1 and TBP, the lowest sample was set to 1 and the mean of HPRT1 and TBP normalized values was used to compare aortas of distinct treatment groups (GSK484, Nox2ds-tat, HIPe and DNase I).

Supplemental Table 1: Frequency of aortic ruptures and intramural thrombus occurrence in the NET inhibitor studies based on the Ang-II model.

**No AAA development at day 8**		**7.2%**
**Rupture before day 10**		**34.5%**
**Mice in treatment (n)**		**68**
**Late ruptures during treatment**		**8**
Treatment	Late rupture day	
PBS	d12, d14	3/33 (9.1%)
GSK484	d11, d13	2/9 (22.2%)
Nox2ds-tat	d16	1/9 (11.1%)
HIPe	d24	1/9 (11.1%)
DNase	d24	1/8 (12.5%)
**Mice completing treatment**		**60**
without thrombus		25/60 (41.7%)
with thrombus		35/60 (58.3%)

In total, 35% of mice suffered from aortic ruptures (abdominal or thoracic) in the first 10 days of AAA induction, while 7% of mice did not reach the defined cut-off of 120% aortic volume growth at day 8 for aneurysm formation and were excluded from the study. The remaining mice (n=68) were stratified into control or treatment groups, but a few late aortic ruptures/deaths were observed. The presence of an intramural thrombus as recorded in ultrasound analysis at day 8 or day 27 was additionally documented.

Supplemental Table 2: Source of primer sequences applied in real-time PCR.

Gene ID	Gene name	Detection method	Primer source
ACTA2	alpha-smooth muscle actin (SMA)	TaqMan™ Assay	Mm01546133_m1
CCL2	monocyte chemotactic protein 1 (MCP-1)	TaqMan™ Assay	Mm00441242_m1
CD68	macrosialin	TaqMan™ Assay	Mm03047343_m1
CNN1	calponin 1	SYBR Green™ Assay	[10]
COL3A1	collagen type III alpha 1	SYBR Green™ Assay	[11]
ELN	elastin	SYBR Green™ Assay	[12]
GPX4	glutathione peroxidase 4	TaqMan™ Assay	Mm04411498_m1
HPRT1	hypoxanthine guanine phosphoribosyl transferase 1	SYBR Green™ Assay	[13]
IL6	interleukin 6	SYBR Green™ Assay	[14]
IFNG	interferon gamma	TaqMan™ Assay	Mm01168134_m1
MMP2	matrix metalloproteinase 2	TaqMan™ Assay	Mm00439498_m1
MMP9	matrix metalloproteinase 9	SYBR Green™ Assay	[15]
MPO	myeloperoxidase	TaqMan™ Assay	Mm01298424_m1
MYH11	myosin (heavy chain) 11	SYBR Green™ Assay	[16]
SLC7A11	solute carrier family 7 member 11	TaqMan™ Assay	Mm00442530_m1
TAGLN	transgelin	SYBR Green™ Assay	[17]
TBP	TATA box binding protein	SYBR Green™ Assay	[18]

Supplemental Figures

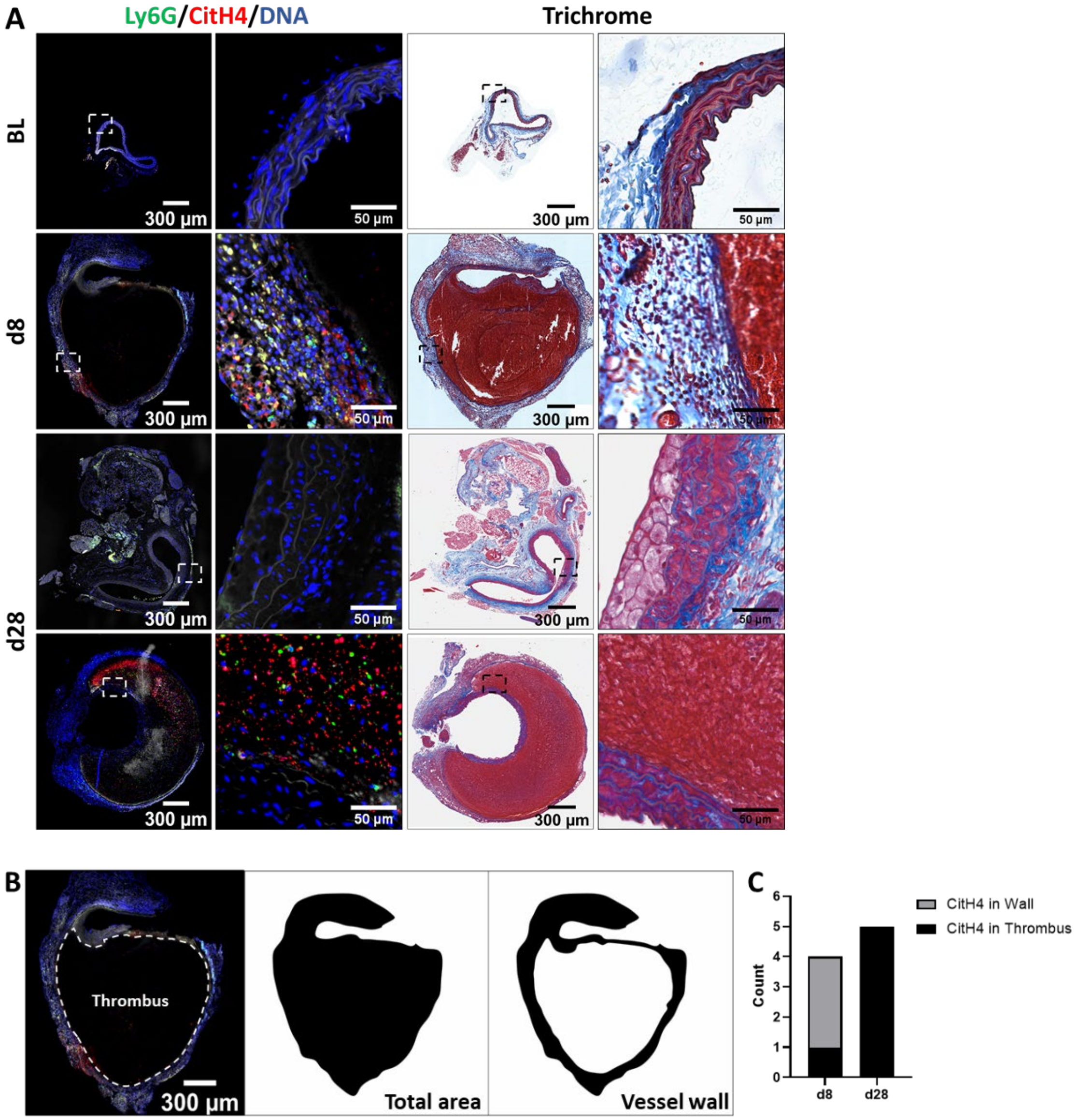

Supplemental Figure 1: Time course of neutrophil extracellular trap (NET) presence in the Ang-II mouse model. (A) Suprarenal aortic sections from baseline (BL), day 8, and day 28 were immunofluorescence stained for NETs: CitH4 (red), Ly6G (green), and DNA (blue), and weak autofluorescence of elastin/collagen fibers is depicted in white. AAA examples without (upper) or with (lower panel) intramural thrombus development are given for day 28. Consecutive sections were also subjected to a Masson's trichrome stain. Sections were scanned using a 20x objective.

The marked areas of interest are shown in the zoomed images. **(B)** A schematic illustration is given for the quantification of NET area (% of area covered by CitH4 signal) which was conducted with CellProfiler and separately calculated for vessel wall (total area - thrombus) and for thrombus area. **(C)** The part (wall or thrombus) that was dominated with CitH4 coverage was denoted. At d8 (n=4), NETs were primarily found in the aortic wall, while at d28 (n=5), all remaining NET signal was detected in the thrombus. Mice without thrombus were excluded from this subgroup analysis. Categorical analysis was performed by Fisher's exact test and yielded p=0.048.

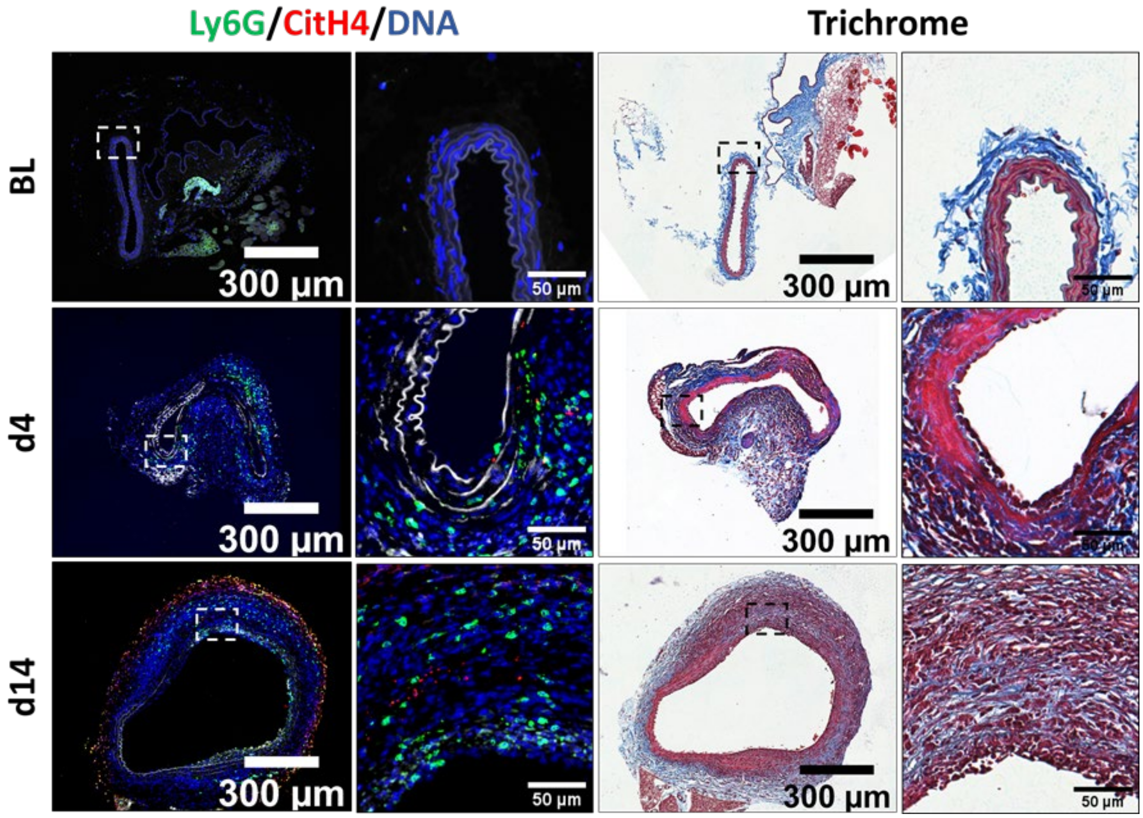

Supplemental Figure 2: Time course of neutrophil extracellular trap (NET) presence in the EPPE mouse model. Infrarenal aortic sections from baseline (BL), day 4, and day 14 were immunofluorescence stained for NETs: CitH4 (red), Ly6G (green) and DNA (blue), and weak autofluorescence of elastin and collagen fibers is depicted in white. Consecutive sections were also subjected to Masson's trichrome stain. Sections were scanned using a 20x objective. The marked areas of interest are shown in the zoomed images. Of note, CitH4 was predominantly detectable in areas where elastin fibers were completely absent/most broken, while areas with intact elastin fibers did not show a signal.

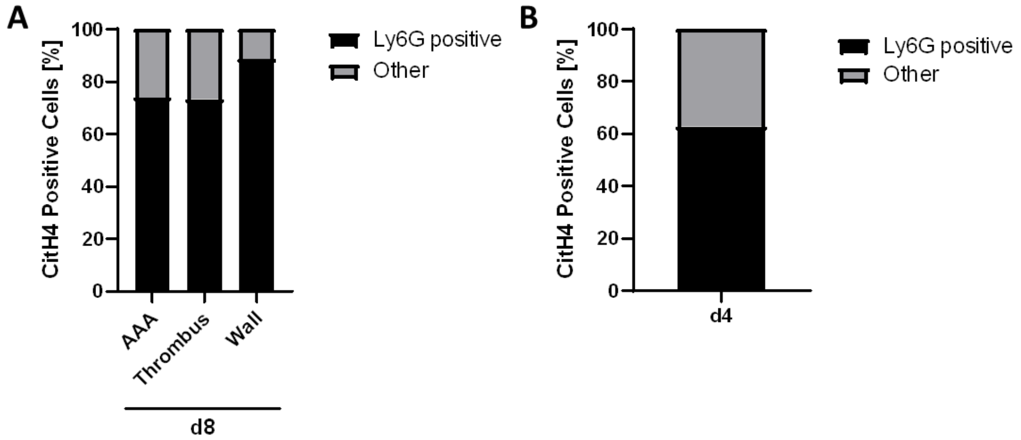

Supplemental Figure 3: Abundance of NET versus other extracellular trap sources in AAA mouse models. Quantification of CitH4 positive cells and co-localization with Ly6G signal was conducted to determine the contribution of neutrophils versus other cell types to extracellular trap formation in **(A)** the Ang-II model at day 8 (for total AAA area or separately for thrombus and vessel wall = total AAA area – thrombus), and in **(B)** the EPPE model at day 4 (n=4/model).

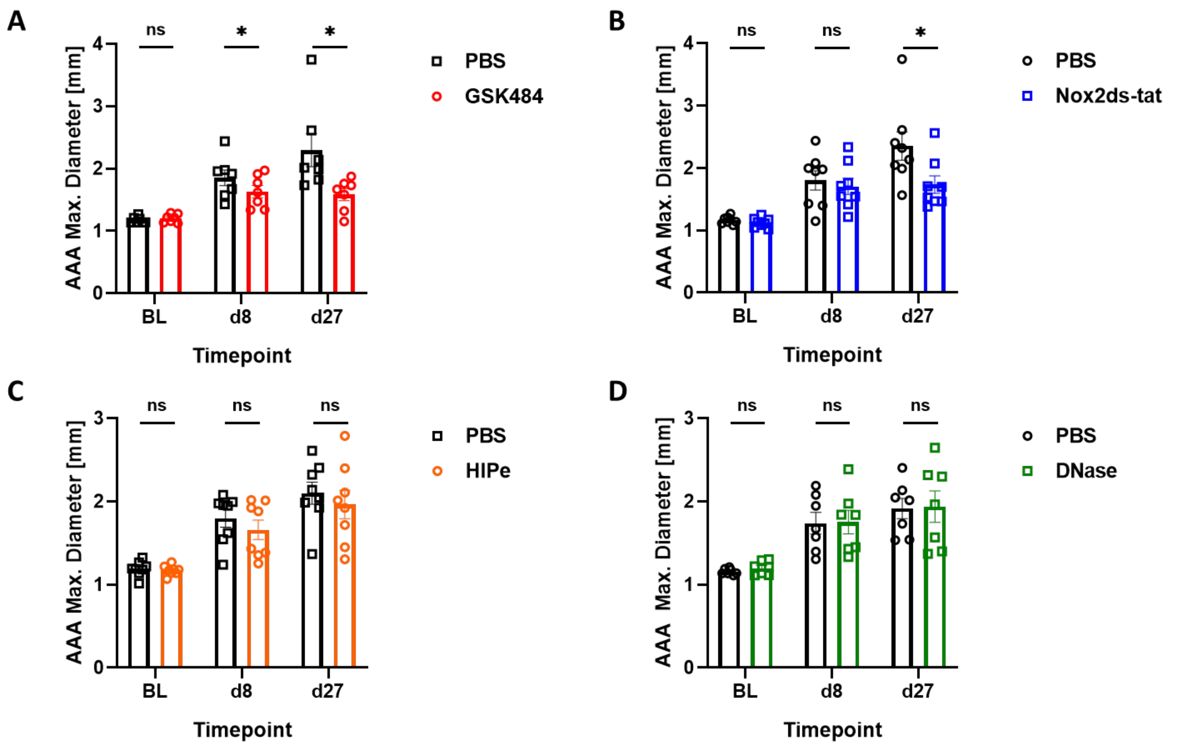

Supplemental Figure 4: Upstream versus downstream NET inhibition in the Ang-II model. AAA size is expressed as maximum aortic diameter [mm]. Mice were matched 1:1 at day 8 with mice from the control (PBS) group by increase in aortic volume percent (in comparison to BL=100%), and treatment was injected intravenously daily from day 9 to day 27. The upstream pathways of NET formation were targeted with **(A)** GSK484 (n=7/group), and **(B)** Nox2ds-tat (n=8/group) treatment. To inhibit the downstream products of NETs, **(C)** HIPe (n=8/group), and **(D)** DNase I (n=7/group) were administered. Data are presented as individual points with mean ± SEM, and differences between groups were evaluated by Wilcoxon signed-rank test with * $p < 0.05$, ns: not significant.

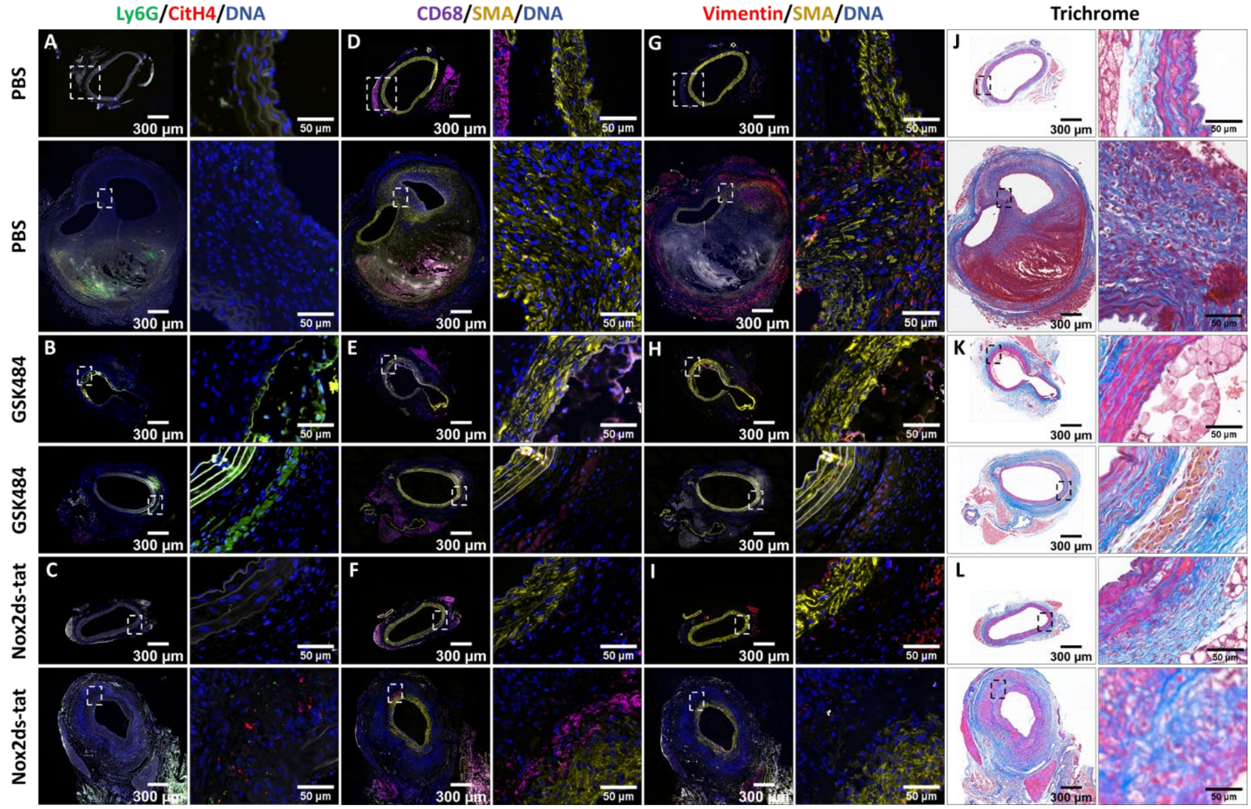

Supplemental Figure 5: NET accumulation in AAA tissue at day 28 after treatment with PBS or upstream inhibitors in the Ang-II model. Suprarenal consecutive aortic sections from day 28 were stained for NETs **(A-C)** by CitH4 (red) and Ly6G (green), while smooth muscle cells and macrophages **(D-F)** were visualized with SMA (yellow) and CD68 (magenta), respectively. Synthetic SMCs **(G-I)** are revealed by double staining of vimentin (red) and SMA (yellow). DNA counterstain is depicted in blue, and weak autofluorescence of elastin and collagen fibers in white. **(J-L)** Samples were also subjected to Masson's trichrome stain. Sections were scanned using a 20x objective. The marked areas of interest are shown in the zoomed images. For each treatment, one aorta without thrombus (upper panel) and one with a thrombus (lower panel) are displayed.

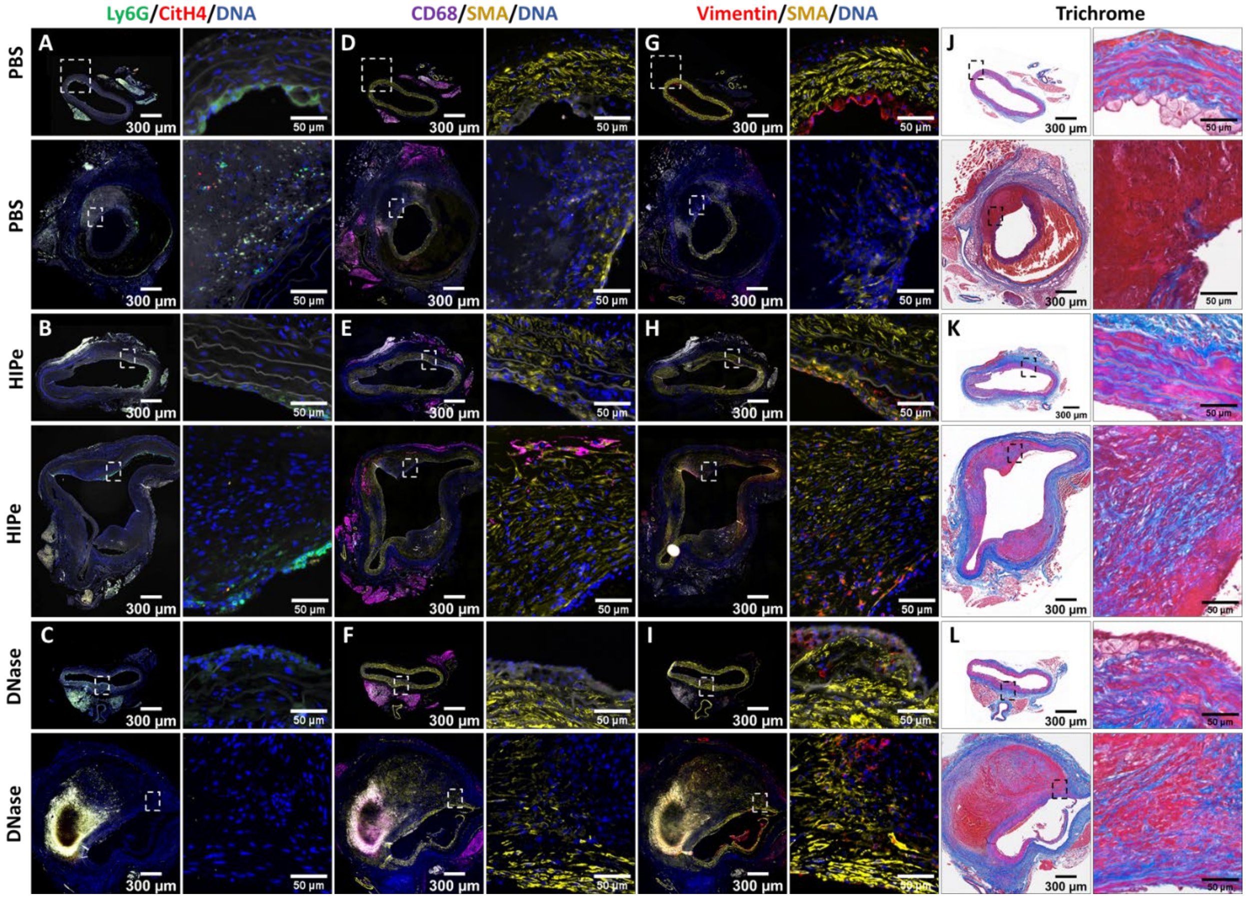

Supplemental Figure 6: NET accumulation in AAA tissue at day 28 after treatment with PBS or downstream inhibitors in the Ang-II model. Suprarenal consecutive aortic sections from day 28 were stained for NETs **(A-C)** by CitH4 (red) and Ly6G (green) immunofluorescence, while smooth muscle cells **(D-F, G-I)** and macrophages **(D-F)**, were visualized with SMA (yellow) and CD68 (magenta), respectively. Synthetic SMCs **(G-I)** are revealed by double staining of vimentin (red) and SMA (yellow). DNA counterstain is depicted in blue, and weak autofluorescence of elastin and collagen fibers in white. **(J-L)** Samples were also subjected to Masson's trichrome stain. Sections were scanned using a 20x objective. The marked areas of interest are shown in the zoomed images. For each treatment, one aorta with no thrombus (upper panel) and one with a thrombus (lower panel) are displayed.

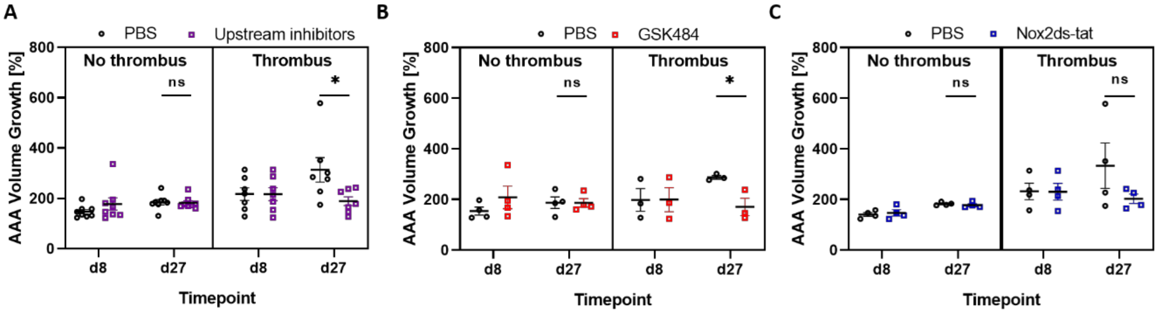

Supplemental Figure 7: Upstream NET inhibition in Ang-II mice without or with AAA-associated thrombus. Mice were divided into subgroups based on intramural thrombus presence in 3D ultrasound evaluation. AAA volume growth was recorded in percent of baseline (100%). **(A)** Data of upstream inhibitors (GSK484 and Nox2ds-tat) were pooled and compared to the control (PBS) group by Wilcoxon rank-sum test at the experimental endpoint. Data of **(B)** GSK484 and **(C)** Nox2ds-tat were separately compared to the PBS-treated mice. Data are presented as individual points with mean ± SEM, and p-values of Wilcoxon rank-sum test at d27 are indicated in the plots: * $p < 0.05$, ns: not significant.

A

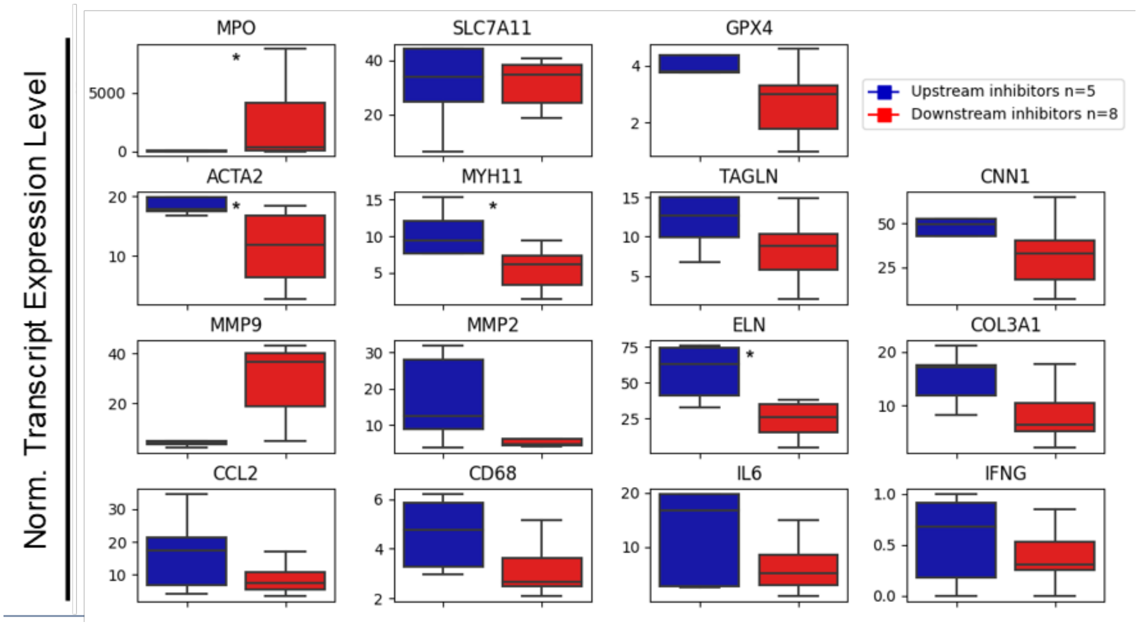

B

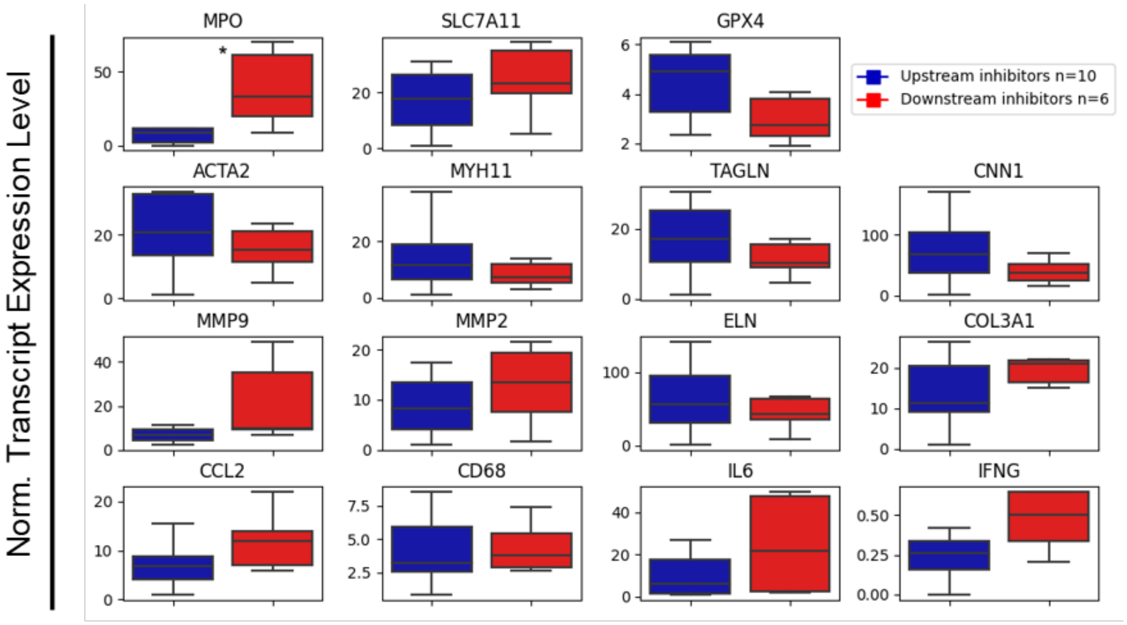

Supplemental Figure 8: Impact of NET inhibitors on mediators of inflammation, matrix remodeling and SMC transdifferentiation in relation to thrombus formation of Ang-II treated mice. Aneurysms from mice exposed to upstream or downstream inhibitors of NET

formation were subjected to RNA isolation, cDNA synthesis and real-time PCR to evaluate transcript levels of genes involved in redox regulation/ferroptosis (MPO, SLC7A11, GPX4), SMC differentiation (ACTA2, MYH11, TAGLN, CNN1), ECM remodeling (MMP9, MMP2, ELN, COL3A1) and inflammation (CCL2, CD68, IL6, IFNG). Please refer to *Supplemental Table 2* for gene symbols, synonyms and qPCR primer sources. Data are presented as boxplots, are shown separately for **(A)** mice with or **(B)** mice without intramural thrombus development (Wilcoxon rank-sum test: * $p < 0.05$).

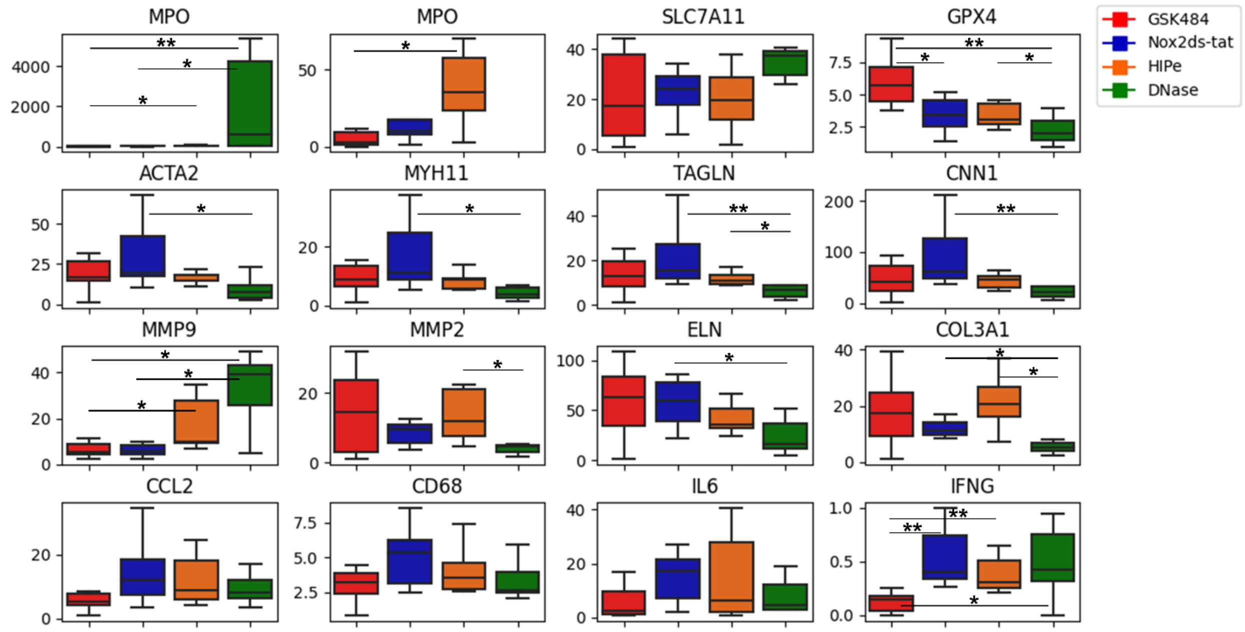

Supplemental Figure 9: Impact of NET inhibitors on mediators of inflammation, matrix remodeling and SMC transdifferentiation in Ang-II treated mice. Aneurysms from mice exposed to GSK484 (n=7), Nox2ds-tat (n=8), HIPe (n=7) or DNase I (n=7) were subjected to RNA isolation, cDNA synthesis and real-time PCR to evaluate transcript levels of genes involved in redox regulation/ferroptosis (MPO, SLC7A11, GPX4), SMC differentiation (ACTA2, MYH11, TAGLN, CNN1), ECM remodeling (MMP9, MMP2, ELN, COL3A1) and inflammation (CCL2, CD68, IL6, IFNG). Please refer to *Supplemental Table 2* for gene symbols, synonyms and qPCR primer sources. Data are presented as boxplots; differences between groups are indicated by * $p < 0.05$, ** $p < 0.01$, *** $p < 0.001$ as evaluated by Wilcoxon rank-sum test.

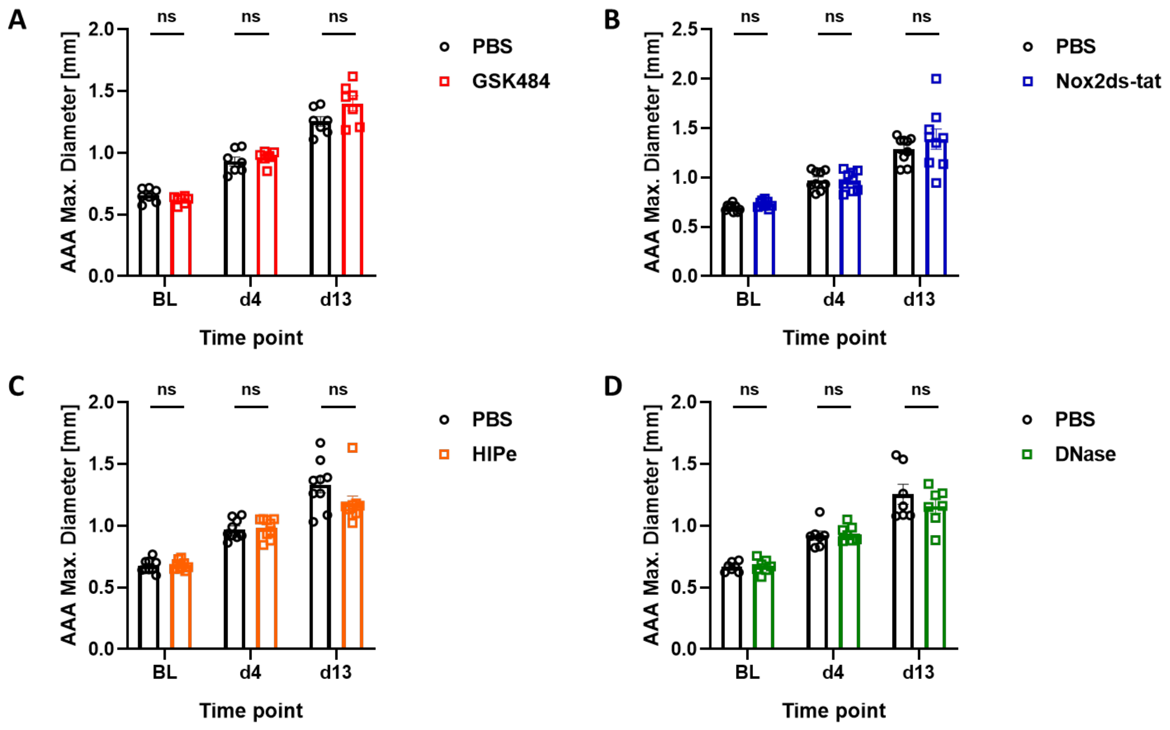

Supplemental Figure 10: Upstream versus downstream NET inhibition in the EPPE model. AAA size is expressed as maximum aortic diameter [mm]. Mice were matched 1:1 at day 4 (d4) with mice from the control (PBS) group by increase in aortic volume percent (in comparison to BL), and treatment was injected intravenously daily from day 5 to day 13. The upstream pathways of NET induction were targeted with **(A)** GSK484 (n=7/group), and **(B)** Nox2ds-tat (n=9/group) treatment. To inhibit the downstream products of NETs, **(C)** HIPe (n=9/group), and **(D)** DNase I (n=7/group) were administered. Data are presented as individual points with mean ± SEM, and differences between groups were evaluated by Wilcoxon signed-rank test with * $p < 0.05$, ns: not significant.

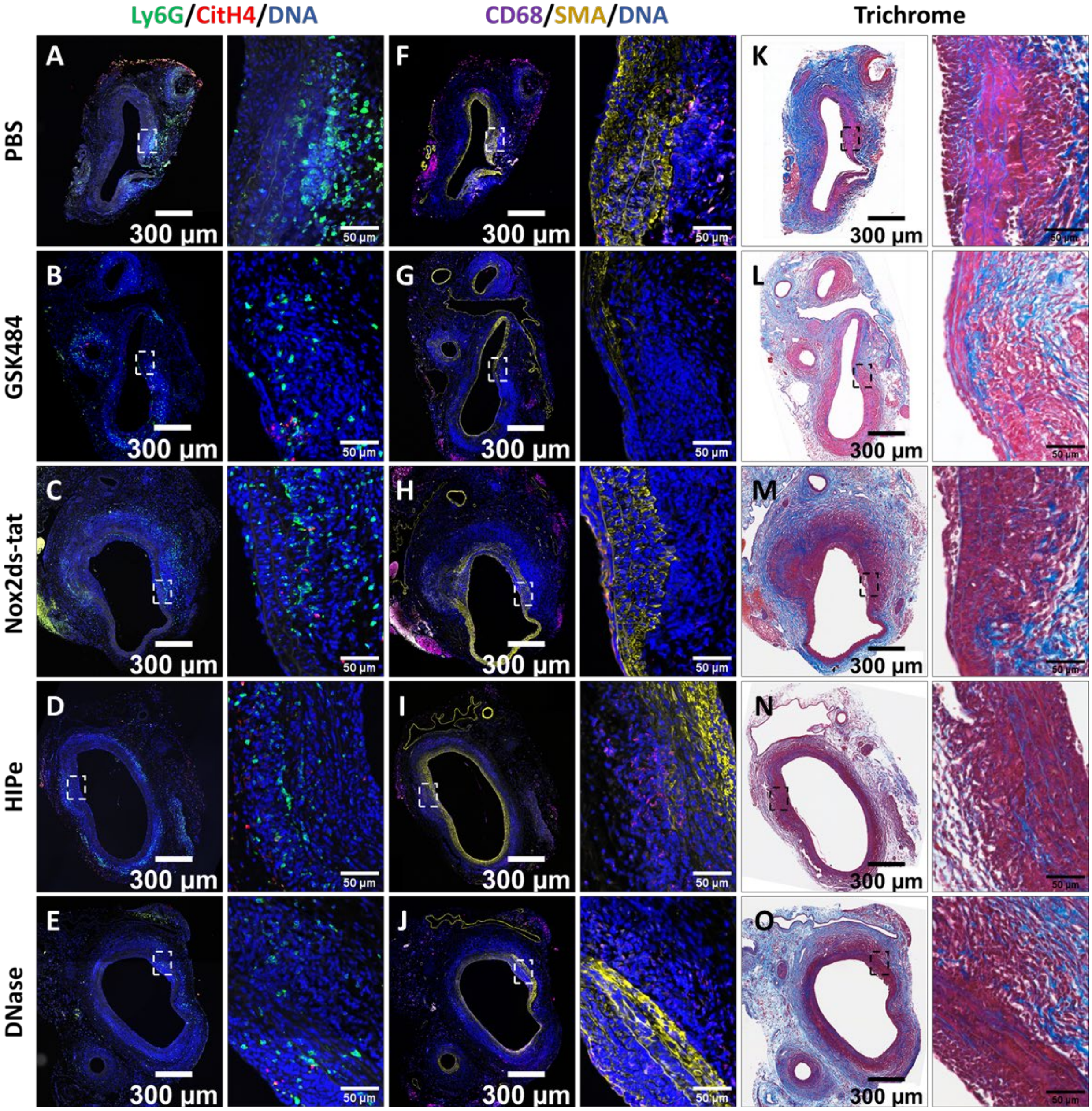

Supplemental Figure 11: NET accumulation in AAA tissue at day 14 after PBS or anti-NET treatment in the EPPE model. Infrarenal consecutive aortic sections from day 14 were stained for NETs **(A-E)** by CitH4 (red), Ly6G (green) and DNA (blue) immunofluorescence, while smooth muscle cells and macrophages **(F-J)** were visualized with SMA (yellow), CD68 (magenta), and DNA (blue) staining; weak autofluorescence of elastin and collagen fibers is depicted in white.

(K-O) Samples were also subjected to Masson's trichrome stain. Sections were scanned using a 20x objective. The marked areas of interest are shown in the zoomed images.

Supplemental References

1. Ibrahim N, Bleichert S, Klopf J, et al. 3D Ultrasound Measurements Are Highly Sensitive to Monitor Formation and Progression of Abdominal Aortic Aneurysms in Mouse Models. *Front Cardiovasc Med*. 2022;9. doi:10.3389/FCVM.2022.944180/FULL

2. Waduud MA, Kandavelu P, Reay M, Paradine K, Scott DJA, Bailey MA. High-Frequency Three-Dimensional Lumen Volume Ultrasound Is a Sensitive Method to Detect Early Aneurysmal Change in Elastase-Induced Murine Abdominal Aortic Aneurysm. *Aorta*. 2020;9(6):215-220. doi:10.1055/s-0041-1731404

3. Eilenberg W, Zagrapan B, Bleichert S, et al. Histone citrullination as a novel biomarker and target to inhibit progression of abdominal aortic aneurysms. *Translational Research*. 2021;233:32-46. doi:10.1016/j.trsl.2021.02.003

4. Lewis HD, Liddle J, Coote JE, et al. Inhibition of PAD4 activity is sufficient to disrupt mouse and human NET formation. *Nat Chem Biol*. 2015;11(3):189-191. doi:10.1038/nchembio.1735

5. Cifuentes-Pagano ME, Meijles DN, Pagano PJ. Nox Inhibitors & Therapies: Rational Design of Peptidic and Small Molecule Inhibitors. *Curr Pharm Des*. 2015;21(41):6023. doi:10.2174/1381612821666151029112013

6. Silvestre-Roig C, Braster Q, Wichapong K, et al. Externalized histone H4 orchestrates chronic inflammation by inducing lytic cell death. *Nature*. 2019;569(7755):236-240. doi:10.1038/s41586-019-1167-6

7. Meher AK, Spinosa M, Davis JP, et al. Novel Role of IL (Interleukin)-1β in Neutrophil Extracellular Trap Formation and Abdominal Aortic Aneurysms. *Arterioscler Thromb Vasc Biol*. 2018;38(4):843-853. doi:10.1161/ATVBAHA.117.309897

8. Bankhead P, Loughrey MB, Fernández JA, et al. QuPath: Open source software for digital pathology image analysis. *Scientific Reports 2017 7:1*. 2017;7(1):1-7. doi:10.1038/s41598-017-17204-5

9. Masuda N, Ohnishi T, Kawamoto S, Monden M, Okubo K. Analysis of chemical modification of RNA from formalin-fixed samples and optimization of molecular biology applications for such samples. *Nucleic Acids Res*. 1999;27(22):4436-4443. doi:10.1093/NAR/27.22.4436

10. Li J, Li SH, Wu J, et al. Young Bone Marrow Sca-1 Cells Rejuvenate the Aged Heart by Promoting Epithelial-to-Mesenchymal Transition. *Theranostics*. 2018;8(7):1766-1781. doi:10.7150/THNO.22788

11. Cavarra E, Fardin P, Fineschi S, et al. Early response of gene clusters is associated with mouse lung resistance or sensitivity to cigarette smoke. *Am J Physiol Lung Cell Mol Physiol*. 2009;296(3). doi:10.1152/AJPLUNG.90382.2008

12. Wei H, Hu JH, Angelov SN, et al. Aortopathy in a Mouse Model of Marfan Syndrome Is Not Mediated by Altered Transforming Growth Factor β Signaling. *J Am Heart Assoc*. 2017;6(1). doi:10.1161/JAHA.116.004968

13. Lee DD, Schwarz MA. Adapted approach to profile genes while reconciling Vegf-a mRNA expression in the developing and injured lung. *Am J Physiol Lung Cell Mol Physiol*. 2015;308(12):1202-1211. doi:10.1152/AJPLUNG.00053.2015

14. Besnard V, Dagher R, Madjer T, et al. Identification of periplakin as a major regulator of lung injury and repair in mice. *JCI Insight*. 2018;3(5). doi:10.1172/JCI.INSIGHT.90163

15. Yen JH, Khayrullina T, Ganea D. PGE2-induced metalloproteinase-9 is essential for dendritic cell migration. *Blood*. 2008;111(1):260-270. doi:10.1182/BLOOD-2007-05-090613

16. Long X, Tharp DL, Georger MA, et al. The smooth muscle cell-restricted KCNMB1 ion channel subunit is a direct transcriptional target of serum response factor and myocardin. *J Biol Chem*. 2009;284(48):33671-33682. doi:10.1074/JBC.M109.050419

17. Wang X, Hu G, Zhou J. Repression of versican expression by microRNA-143. *J Biol Chem*. 2010;285(30):23241-23250. doi:10.1074/JBC.M109.084673

18. Ho KH, Patrizi A. Assessment of common housekeeping genes as reference for gene expression studies using RT-qPCR in mouse choroid plexus. *Sci Rep*. 2021;11(1). doi:10.1038/S41598-021-82800-5
